# An AI-powered knowledge hub for potato functional genomics

**DOI:** 10.1016/j.xplc.2026.101730

**Published:** 2026-01-14

**Authors:** Jinye Li, Yudong Jia, Futing Li, Xiaoqi Su, Jilin Luo, Yarui Dong, Chunyan Sun, Qinghan Cui, Li Wang, Axiu Li, Yi Shang, Sanwen Huang, Yujuan Zhu, Yuxin Jia

**Affiliations:** 1 Engineering Research Center for Valorization of Unique Bio-Resources in Yunnan, Ministry of Education, School of Life Sciences, Yunnan Normal University, Kunming 650500, China; 2 Shenzhen Branch, Guangdong Laboratory of Lingnan Modern Agriculture, Genome Analysis Laboratory of the Ministry of Agriculture and Rural Affairs, Agricultural Genomics Institute at Shenzhen, Chinese Academy of Agricultural Sciences, Shenzhen, Guangdong 518120, China; 3 Southwest United Graduate School, Kunming 650092, China; 4 School of Information and Communication Engineering, Beijing University of Posts and Telecommunications, Beijing 100876, China

Dear Editor,

Potato is one of the most important tuber crops, feeding approximately 1.3 billion people worldwide. Functional gene research is fundamental for enhancing crop yield, quality, and resilience to the rapidly changing global climate (Wang et al., 2025). Potato functional genomics has advanced significantly in recent years (Deng et al., 2024; Plunkert et al., 2024; Lian et al., 2025). However, iterative updates to the potato reference genome (DM1-3 516 R44) have introduced incompatible gene nomenclature systems, creating persistent challenges in reconciling gene IDs and symbols across historical studies (Supplemental Figure 1). In parallel, an ∼100% increase in related publications over the past decade has overwhelmed conventional literature mining methods (Supplemental Figure 2). Collectively, these issues are hindering progress in potato functional genomics. The recent emergence of large language models (LLMs) represents a paradigm-shifting advance in processing exabyte-scale heterogeneous data (Yang et al., 2025; Zhang et al., 2025). Subsequently, AI agent systems have been developed to extend LLMs’ capabilities from cognitive assistance (“knowing how”) to operational autonomy (“getting it done”), creating transformative opportunities for addressing data-intensive challenges across diverse biological research domains.

To leverage LLMs and AI agents for advancing potato research and to demonstrate their potential in functional genomics, we present the Potato Knowledge Hub (https://www.potato-ai.top; Figure 1), a freely accessible platform for the potato research community. This integrated platform comprises three key modules: (1) a potato knowledge database powered by a retrieval-augmented generation (RAG) architecture, drawing from over 6112 high-quality potato research articles, which overcomes key limitations of general LLMs (static knowledge and hallucination risk) to provide accurate scientific responses; (2) a manually curated functional gene database based on the latest potato genome assembly, featuring 2853 reported functional genes with cross-version ID mapping; and (3) a practical toolkit incorporating BLAST, ID conversion, gene annotation, and Gene Ontology/Kyoto Encyclopedia of Genes and Genomes enrichment analyses for efficient data processing. Moreover, we developed the Potato Research Assistant, an AI agent that integrates all platform modules to facilitate intuitive, natural language-based interaction. The Potato Knowledge Hub is designed to provide a streamlined user experience, freeing researchers from labor-intensive literature mining and fragmented analytical workflows. By substantially enhancing individual research efficiency, this platform promises to accelerate discoveries across the field of potato functional genomics.

**Figure 1:**
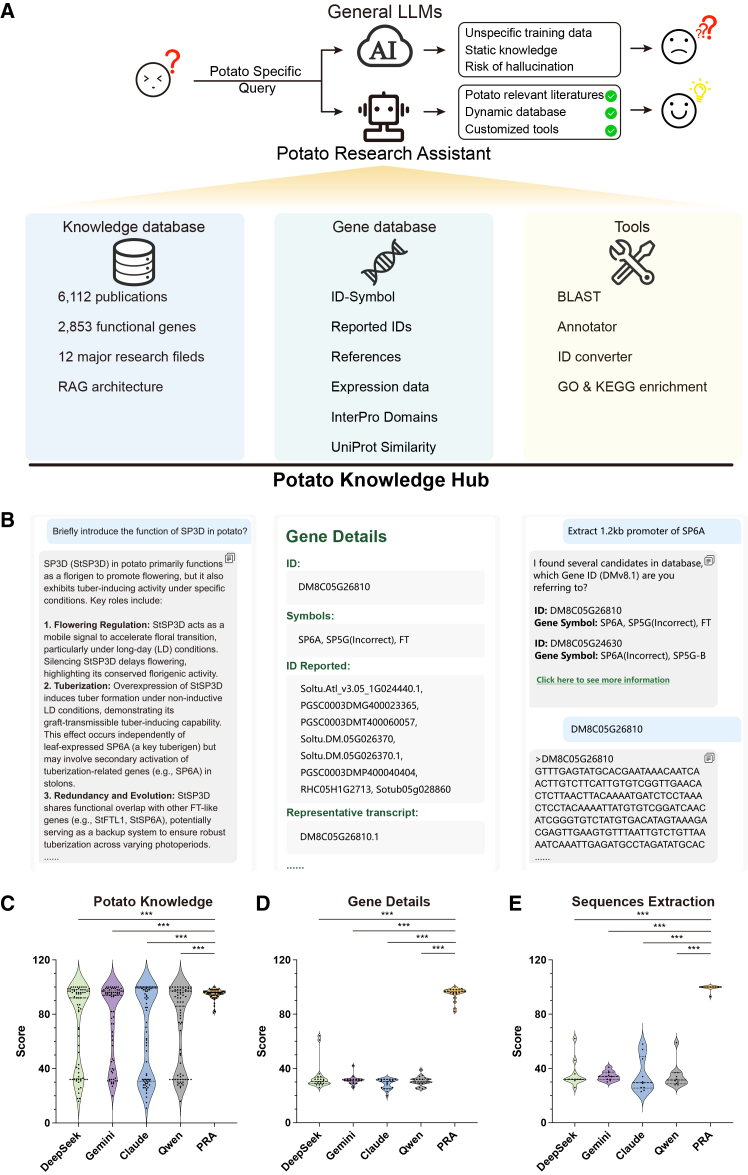
Architecture and performance evaluation of the Potato Knowledge Hub. **(A)** Schematic representation of the Potato Knowledge Hub system architecture. RAG, retrieval-augmented generation. **(B)** Overview of the functional modules of the Potato Knowledge Hub. **(C–E)** Performance comparison between the Potato Research Assistant (PRA) and general LLMs in **(C)** potato knowledge queries, **(D)** gene detail comprehension, and **(E)** sequence extraction. Statistical significance was assessed using Student’s *t*-test. ∗∗∗*P* < 0.001.

The first step in developing the Potato Knowledge Hub was to construct a high-quality, specialized potato research corpus. To comprehensively capture potato research, we searched NCBI PubMed and the Web of Science Core Collection using “potato” as a keyword in titles or abstracts. This search, spanning from January 1, 1900, to the present, retrieved over 57 795 nonredundant papers (Supplemental Figure 2). To enrich for high-quality studies, we consulted the Chinese Academy of Sciences journal ranking and extracted research articles published in two top-tier journal categories (Q1 and Q2) in biology, agriculture, and forestry sciences and multidisciplinary categories, yielding 21 505 papers. We then implemented LLMs to analyze titles and abstracts and to exclude studies that only mentioned “potato” without using potato as the research subject. This screening yielded 6007 studies relevant to potato breeding, genetics, metabolism, physiology, genomics, and functional gene research. We further manually reviewed report- or letter-type papers lacking abstract information and retrieved 105 more papers. To assess database coverage, we manually reviewed the 100 most recent publications in potato functional gene research and confirmed that all were included. In total, we collected 6112 high-quality potato research papers. To ensure detailed and accurate content, we downloaded full-text Portable Document Formats, rather than relying only on abstracts. Each Portable Document Format was converted to plain text for downstream data processing (Supplemental Figure 3).

Paragraphs from each paper were analyzed using an LLM to exclude nonscientific sections, including author information, affiliations, references, and acknowledgments. A RAG architecture was applied to enhance response accuracy and reduce hallucinations. The full text of each paper was segmented and vectorized using an embedding model. To support multi-turn interactions, an LLM analyzed the user query in its conversational context and rephrased it to improve knowledge retrieval efficiency. The rephrased query was vectorized using the same model, and the top 200 relevant paragraphs were retrieved based on embedding distance. A re-ranking model then rescored semantic relevance between each segment and the query. The highest-ranking segments, together with the user query, were submitted to the LLM to generate the final answer (Supplemental Figure 4A and 4B).

To determine the optimal chunking and retrieval settings that maximize performance, we evaluated three chunking methods (100 words with 60-word overlap, 200 words with 120-word overlap, and paragraph-based chunking) combined with three retrieval depths (top 5, 10, and 20 chunks after re-ranking). We collected 70 potato knowledge questions and corresponding validated answers provided by professors, postdoctoral researchers, and graduate students. We then employed an “LLM as a judge” framework, which provides a scalable and interpretable proxy for human evaluation (Zheng et al., 2023). Specifically, a third-party LLM (GPT-o3) acted as the judge and scored responses against the validated answers across four dimensions: knowledge accuracy (40 points), completeness (30 points), relevance (20 points), and clarity (10 points).

The results indicate that paragraph-based segmentation yields higher and more concentrated scores. Conversely, fixed-length segmentation exhibits a noticeably dispersed score distribution across all retrieval depths even though the evaluation scores obtained with 200-word segmentation did not differ significantly from those of paragraph-based segmentation. Under paragraph-based segmentation, retrieving the top 5 segments led to incomplete answers for some questions because of insufficient reference content, resulting in scores below 70. The score distributions obtained when retrieving the top 10 or top 20 natural paragraphs were highly similar (Supplemental Figure 4C). Consequently, we adopted paragraph-based text segmentation and retrieval of the top 10 segments for LLM-based answer generation. This curated potato research corpus, combined with the RAG-based framework, enables precise, domain-specific answers with minimal hallucination risk (Figure 1B and 1C and Supplemental Figure 5).

The continuously updated potato reference genomes, together with incompatible gene nomenclature systems, pose a persistent challenge for reconciling gene IDs and symbols across historical studies (Potato Genome Sequencing et al., 2011; Pham et al., 2020; Yang et al., 2023) (Supplemental Figure 1). To address this issue, we developed an LLM-driven gene entity normalization workflow to accurately map previously reported genes to the latest genome assembly (DMv8.1). An LLM was employed to analyze the full plain text of each paper and extract reported functional gene symbols and corresponding IDs. Two independent rounds of extraction were performed using different LLMs to maximize coverage. The merged results were then subjected to manual curation, yielding 4947 gene ID–symbol pairs. These gene IDs originated from multiple databases and genome versions, including GenBank, UniProt, Solanaceae Genomics Network, ATL_v3, SolTub3.0, DMv4.03, DMv6.1, and DMv8.1. All identifiers were mapped to the DMv8.1 reference genome using genome synteny and BLAST-based sequence identity. This process resulted in a final set of 2853 non-redundant functional genes (Supplemental Figure 6A). During manual curation, we also identified and corrected historical nomenclature errors (for example, SP5G-B being mislabeled as SP6A; Herrera-Isidron et al., 2024), with all corrections annotated and traceable in our database. To ensure full transparency, our database maintains direct links connecting DMv8.1 gene IDs, legacy identifiers, and gene symbols associated with their respective source publications. To further enhance research utility, we integrated key functional annotations, including InterPro domains, UniProt similarity information, and expression patterns, into each gene entry (Figure 1B and Supplemental Figure 6B). We also developed a toolkit comprising BLAST, Annotator, ID converter, and Gene Ontology and Kyoto Encyclopedia of Genes and Genomes enrichment tools to facilitate rapid BLAST queries, gene domain and symbol annotation, DMv4 and DMv6 to DMv8 ID conversion, and functional enrichment analyses (Supplemental Figure 7). Together, this gene database enables researchers to retrieve comprehensive gene information and associated literature using either current gene symbols or legacy identifiers, significantly simplifying the background research process for functional gene studies and facilitating efficient project initiation.

To optimize user interaction with the Potato Knowledge Hub, we implemented an agentic task orchestration architecture and developed the Potato Research Assistant, an AI agent capable of invoking the workflows of the knowledge database, gene database, and integrated toolkit (Figure 1A). This agent interacts with users via natural language, conducts intent analysis, and calls the appropriate internal scripts to summarize potato research knowledge, retrieve gene information and sequences, and execute specific analytical tools. For a queried gene symbol, the assistant can summarize historical research, extract sequences (coding, protein, and customized promoter regions, etc.), and provide detailed information on putative gene function. By integrating these capabilities, the AI agent liberates users from tedious literature mining and complex bioinformatics software, enabling scientists without specialized computational expertise to efficiently explore and utilize a tremendous amount of gene information with ease.

To evaluate the performance of the Potato Research Assistant, in addition to the 70 questions used to benchmark the knowledge base, we collected an additional 20 questions focused on gene understanding and 10 questions related to sequence extraction to assess the gene database. We compared the responses generated by the Potato Research Assistant with those produced by state-of-the-art general LLMs; specifically, the open-source models DeepSeek-R1-0528 and Qwen3-235B-A22B (thinking enabled) as well as the closed-source models Gemini 2.5 Pro Preview-0506 and Claude Sonnet 4. Responses from both the Potato Research Assistant and the general LLMs were evaluated using the same scoring system applied in the previous RAG evaluation. The Potato Research Assistant outperformed the general LLMs across all three evaluation categories. This performance advantage underscores the effectiveness of our agentic task orchestration architecture and specialized database design in enabling superior domain expert knowledge mastery compared with general-purpose LLMs. For knowledge retrieval tasks, the assistant showed higher performance stability, whereas general LLMs exhibited score polarization, likely due to an incomplete training corpus (Figure 1C and Supplemental Figure 5). For gene detail comprehension and sequence extraction, the assistant demonstrated clear advantages, supported by its dedicated potato gene database (Figure 1D and 1E and Supplemental Figures 8 and 9).

Our work addresses key challenges in potato functional genomics research, which is representative of crops with high genetic complexity and frequent genome version inconsistencies. The Potato Knowledge Hub will be actively updated on a monthly basis to integrate the latest advances in potato research. We anticipate that this hub will serve as a valuable community resource for advancing potato functional genomics and providing crucial information to support potato breeding. Furthermore, the technical strategies developed here, including the LLM-driven gene entity normalization workflow and the agentic task orchestration architecture, offer a transferable framework for developing similar platforms in other areas of biological research.

## Data and code availability

Transcriptome data were obtained from NCBI (BioProject: PRJNA573826). The code for the Potato Knowledge Hub is hosted on Gitee (https://gitee.com/yuxin_2023/potato-knowledge-hub). Benchmark questions, manually curated reference answers, model responses, and evaluation criteria are available on Figshare (https://doi.org/10.6084/m9.figshare.29815859). The UniRef100 database was downloaded from the UniProt FTP server (https://ftp.uniprot.org/).

## Funding

This work was supported by the Guangdong Major Project of Basic and Applied Basic Research (2021B0301030004), the 10.13039/501100001809National Natural Science Foundation of China (32488302, 32302582, and 32402608), the 10.13039/501100012421Agricultural Science and Technology Innovation Program (CAAS-ZDRW202404), the Yunnan Fundamental Research Projects (grants 202501BC070003 and 202501AT070005), the 10.13039/501100002858China Postdoctoral Science Foundation (BX20200376), and the Yunnan Province Xingdian Talent Support Program.

## Acknowledgments

We acknowledge technical support from the Intelligent Computing Center of Yunnan Normal University. We thank Shuhua Yang (China Agricultural University) and Dawei Li, Pei Wang, and Pingxian Zhang (Chinese Academy of Agricultural Sciences) for valuable suggestions that improved the Potato Knowledge Hub. We also thank all researchers who participated in platform testing for performance and stability and who provided constructive feedback. No conflict of interest is declared.

## Author contributions

S.H., Y.Z., and Yuxin Jia conceived the study. Yuxin Jia and Y.Z. wrote the manuscript. Yudong Jia, Yuxin Jia, and J. Li developed the code for the Potato Knowledge Hub. J. Li performed the bioinformatics analyses. Yuxin Jia, Y.Z., J. Li, F.L., X.S., J. Luo, Y.D., C.S., Q.C., L.W., and A.L. collected literature, manually curated gene IDs and symbols, and tested the functionality of the Potato Knowledge Hub. S.H., Y.S., Yuxin Jia, and Y.Z. discussed and revised the manuscript. All authors read and approved the final version.

## Supplemental information

Document S1. Supplemental Figures 1–9 and Supplemental Methods

Document S2. Article plus supplemental information
