# Supplementary material for: An AI-powered knowledge hub for potato functional genomics
Source: Plant Commun. 2026 Jan 14;7(5):101730. doi: 10.1016/j.xplc.2026.101730 (PMC13174205; doi:10.1016/j.xplc.2026.101730)
Supplement: Document S1. Supplemental Figures 1–9 and Supplemental Methods [file mmc1.pdf]

Plant Communications, Volume 7

Supplemental information

An AI-powered knowledge hub for potato functional genomics

Jinye Li, Yudong Jia, Futing Li, Xiaoqi Su, Jilin Luo, Yarui Dong, Chunyan Sun, Qinghan Cui, Li Wang, Axiu Li, Yi Shang, Sanwen Huang, Yujuan Zhu, and Yuxin Jia

Supplementary Information

Title: An AI-powered Knowledge Hub for Potato Functional Genomics

Authors:

Jinye Li[1,3], Yudong Jia[4], Futing Li[1,3], Xiaoqi Su[1,3], Jilin Luo[1,3], Yarui Dong[1], Chunyan Sun[1,3], Qinghan Cui[1,3], Li Wang[1], Axiu Li[1,3], Yi Shang[1,3], Sanwen Huang[2], Yujuan Zhu[2,*], Yuxin Jia[1,3,*]

Affiliations

[1]Engineering Research Center for Valorization of Unique Bio-Resources in Yunnan, Ministry of Education, School of Life Sciences, Yunnan Normal University, Kunming, 650500, China.

[2]Shenzhen Branch, Guangdong Laboratory of Lingnan Modern Agriculture, Genome Analysis Laboratory of the Ministry of Agriculture and Rural Affairs, Agricultural Genomics Institute at Shenzhen, Chinese Academy of Agricultural Sciences, Shenzhen, Guangdong 518120, China.

[3]Southwest United Graduate School, Kunming 650092, China.

[4]School of Information and Communication Engineering, Beijing University of Posts and Telecommunications, Beijing, 100876, China.

[*]Correspondence to: jiayuxin@ynnu.edu.cn (Y.J.), zhuyujuan@caas.cn (Y.Z.)

Contents

METHODS

Fig. S1. Examples of inconsistent gene nomenclature systems across different versions of the potato reference genome.

Fig. S2. The number of publications on potato research has increased significantly in recent years.

Fig. S3. Pipeline of literature processing.

Fig. S4. Development of potato knowledge database.

Fig. S5. Comparison of potato knowledge queries between Potato Research

Assistant and general LLMs.

Fig. S6. Construction of potato gene database.

Fig. S7. Toolkit integrated in Potato Knowledge Hub.

Fig. S8. Comparison of gene detail comprehension between Potato Research Assistant and general LLMs.

Fig. S9. Comparison of sequence extraction between Potato Research Assistant and general LLMs.

METHODS

Processing literatures

Potato-related literature was retrieved by searching the titles or abstracts from the Web of Science Core Collection and PubMed, with "Potato" as the keyword. Relevant studies, including their titles, abstracts, journal information, and DOIs, were exported for subsequent filtering. Papers published in Q2 or higher-ranked journals, according to the CAS Journal Ranking (2023), were retained as high-quality literature. Furthermore, the title and abstract of each paper were then submitted to a LLM to screen out papers that merely mentioned "potato" in the title or abstract but were not focused on potato research fields. The screening was performed twice using DeepSeek-R1-0528 (671B) and Qwen3-235B-A22B (enable thinking), separately. To retain studies more comprehensively, we kept studies that were present in the results from either screen. Literature lacking abstract information underwent further manual review. This collection was designated as high-quality potato literature. Full PDFs of these selected studies were downloaded, and their plain text was extracted using MinerU (v1.2.2), preserving the original texts organization (Wang et al., 2024).

Potato literature knowledge database construction

To improve the quality of data retrieval, we performed data cleaning to remove the text that was not related to the scientific content in the literature. We submitted each paragraph of an article to Qwen3-235B-A22B to filter out authors, affiliations, references, acknowledgements, standalone section titles, page headers, footers, as well as formulas. The vectorization model "text-embedding-v4", developed by the Qwen3 group, was employed to vectorize the collected literature into 2048 dimensions. Each paragraph was treated as an individual chunk for this process. The vectorized data, original text and DOI were stored in Chroma database (1.0.13, https://github.com/chroma-core/chroma).

For data retrieval, the DeepSeek-V3-250324 model was used to rephrase and optimize user's query for vector retrieval, for example, understand user intent based on

multi-turn conversation contexts, add abbreviations to full form phrases or expand abbreviations. The query was vectorized using the same parameters and used to search the Chroma database using the query() function. The top 200 chunks exhibiting the highest similarity scores were extracted. To further identify the most relevant chunks, the reranking model BGE-reranker-v2-m3 was utilized to assess the relevance between these retrieved chunks and the query. The user's query, along with the top relevant chunks, was then sent to the Qwen3-235B-A22B (enable thinking) for summarization.

Potato gene database construction

To extract gene IDs and their corresponding symbols, the plain text of each paper was submitted to DeepSeek-R1-0528 (671B) and Qwen3-235B-A22B (enable thinking) for analysis separately. The resulting data were subsequently merged. All gene ID and symbol correspondences were then manually verified to correct potential inaccuracies. To anchor reported gene IDs to the DMv8.1 reference genome, pairwise synteny analyses were conducted between DMv8.1 and other potato genomes (RH89-039-16, ATL_v3, Jan v1.1, SolTub3.0, DMv4.03, and DMv6.1) using MCscan within the JCVI toolkit (v1.3.6) to identify orthologs (Tang et al., 2024). For genes where syntenic orthologs could not be identified, and for gene sequences sourced from the Sol Genomics Network, UniProt, NCBI GenBank, and the Agria genome, orthologs in DMv8.1 were identified using the NCBI BLAST+ toolkit (v2.16.0) with default parameters and a sequence identity threshold of >90%. Finally, gene IDs, symbols, reported IDs, and their corresponding reference papers were compiled into an SQLite database for efficient data querying.

InterPro domain annotation, uniport similarity and expression analysis of each gene

For comprehensive functional domain annotation and expression analysis of protein-coding genes in the DMv8.1 genome, we performed the analysis in the following steps. First, protein sequences (with stop codons removed) were analyzed using InterProScan (v5.74-105.0) to identify functional domains, and the corresponding InterPro (IPR)

identifiers and domain descriptions were extracted (Jones et al., 2014). In parallel, homology searches were conducted using blastp (v2.14.0+) against the UniRef100 database, retaining only high-confidence matches (E-value < 1×10^{-10}) and selecting the top five hits with the highest sequence identity for functional inference (Suzek et al., 2015). For transcriptomic profiling, publicly available potato RNA-seq datasets were retrieved from the NCBI SRA database (PRJNA573826). Raw sequencing reads were processed for quality control using fastp (v0.24.0), then mapped to the DMv8.1 reference genome using HISAT2 (v2.1.0). Transcript abundance was quantified using StringTie (v2.2.3) and normalized as Transcripts Per Million (TPM) (Chen, 2023; Kim et al., 2015; Pertea et al., 2016). For samples with biological replicates, the mean TPM value across replicates was used to represent the final expression level of each gene, ensuring robust quantification.

AI agent and Potato knowledge hub construction

The "Potato Research Assistant" agent is powered by the Doubao-seed-1.6 LLM (with thinking enabled), which was accessed directly via its API (https://www.volcengine.com/). We employed the model as a frozen reasoning engine without conducting any further continuous pre-training or fine-tuning. The model acts as a reasoning engine designed to analyze user intent via natural language understanding, invoke external databases or tools for information retrieval, and synthesize the final answer based on the context. The Potato Knowledge Hub was developed using the Flask framework (v3.1.0, https://flask.palletsprojects.com/). Python was used to write the backend logical scripts, while HTML and JavaScript were employed for developing the web-based user interface.

API usage and research costs

The APIs for the DeepSeek and Doubao models were provided by VolcanoArk (https://www.volcengine.com/), while the Qwen3 model and the vectorization model text-embedding-v4 were supported by Alibaba Cloud (https://www.aliyun.com/). The API for the re-ranking model BGE-reranker-v2-m3 was provided by SiliconFlow

(https://cloud.siliconflow.cn/me/models). For research costs, constructing the literature knowledge base and functional gene database comprising 6,112 papers consumed approximately 50.1 M tokens for literature screening, 107.6 M tokens for text vectorization, and 2.0 G tokens for extracting gene ID and symbol correspondences.

Tools development

The BLAST tool was developed based on the NCBI BLAST+ toolkit (v2.16.0) (Camacho et al., 2009). The Annotator and ID converter were built using local Python scripts. Gene Ontology (GO) annotations were retrieved in OBO format from the OBO Foundry (http://purl.obolibrary.org/obo/go/go-basic.obo), while KEGG pathway annotations were extracted from the built-in TBtools.KeggBackEnd database in TBtools-II (Chen et al., 2023). The gene annotation file for the DMv8.1 genome was generated using the online platform eggNOG-mapper (http://eggnog-mapper.embl.de/) with default parameters. After establishing the correspondence between the annotation file and the background files, functional enrichment analysis was performed using the enricher function from the R package ClusterProfiler (v4.12.6), with the significance threshold set to pvalueCutoff = 0.05 and adjusted using the Benjamini–Hochberg correction (Wu et al., 2021). The enrichment results were visualized using the ggplot2 package (v3.5.2) (Wickham, 2016).

Performance evaluation

One hundred questions covering potato knowledge queries (70), gene detail comprehension (20), and sequence extraction (10) were collected from professors, postdocs, and graduate students, and the corresponding reference answers were generated and curated manually. We defined a 100-point assessment system consisting of four dimensions: knowledge accuracy (maximum 40 points), completeness (maximum 30 points), relevance (maximum 20 points) and clarity (maximum 10 points). We also carefully defined the judgment rules for each dimension. The LLM-as-a-judge framework was developed based on the reasoning model GPT-o3 (with reasoning_effort set to "high"). The questions, verified answers, meticulous judgment

rules, as well as the corresponding answers from the LLMs and Potato Research Assistant were submitted to GPT-o3 for scoring. Although the LLM-as-a-Judge framework offers scalability, we acknowledge that LLMs may not be entirely impartial and can be susceptible to biases inherent in their training or prompting (Krumdick et al., 2025; Szymanski et al., 2025). To mitigate these potential limitations, we utilized strictly defined judgment rules and expert-validated standard answers as the ground truth for all evaluations. The complete benchmark dataset—benchmark questions, manually curated standard answers, responses from both the Potato Research Assistant and general LLMs, judgment criteria, individual scores, and brief comments explaining each response and score—has been deposited in Figshare (https://doi.org/10.6084/m9.figshare.29815859).

Comparison of performance across different chunking strategies and retrieval counts

To evaluate the impact of different chunking strategies and retrieval depth on LLM generation performance, we compared three text segmentation granularities: fixed window chunking with 100 words/60-word overlap, 200 words/120-word overlap, and paragraph-based chunking. These were cross-tested with three retrieval depth after reranking (Top 5, 10, and 20), resulting in a total of nine test combinations. We selected 70 potato knowledge queries from the above benchmark dataset and employed the "LLM-as-a-judge" framework to automatically score the generation results for each combination. Detailed scoring data have been deposited in Figshare (https://doi.org/10.6084/m9.figshare.29815859).

SolTub_3.0	PGSC DM v4.03	DM v6.1	DM v8.1	Gene Symbol
NM_001287968.1	PGSC0003DMG400023365	Soltu.DM.05G026370	DM8C05G26810	SP6A
LOC102603307	PGSC0003DMG400018408	Soltu.DM.05G005140	DM8C05G04930	CDF1
NM_001288693.1	PGSC0003DMG400004054	Soltu.DM.06G025210	DM8C06G24820	IT1/BRC1b

Fig. S1. Examples of inconsistent gene nomenclature systems across different versions of the potato reference genome.

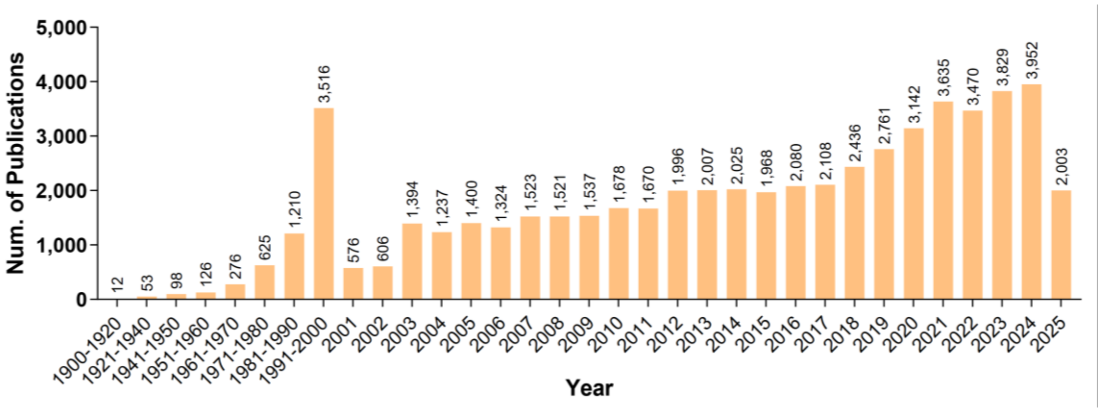

Fig. S2. The number of publications on potato research has increased significantly in recent years.

The dataset was compiled from the Web of Science and PubMed databases using "potato" as a search term in titles or abstracts, with a data retrieval cutoff of June 30, 2025.

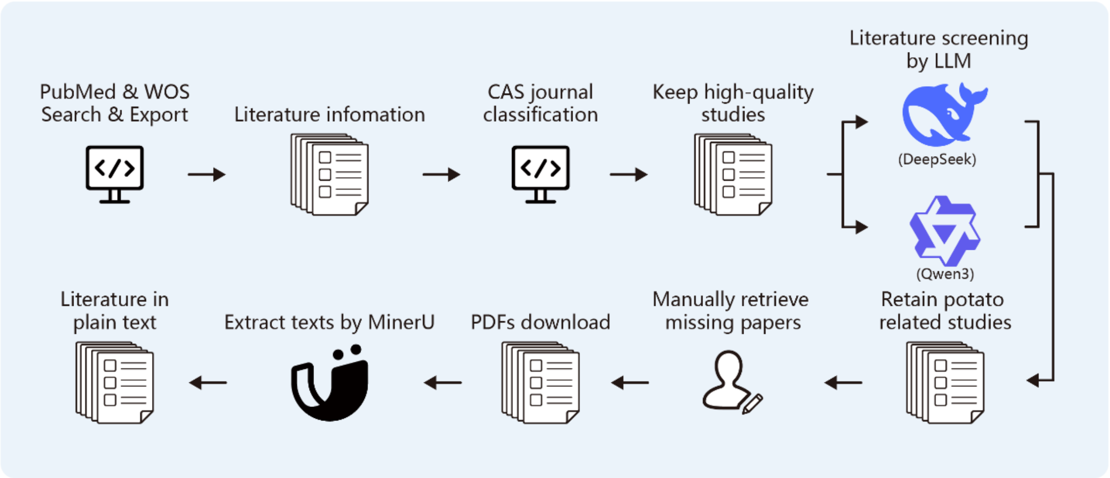

Fig. S3. Pipeline of literature processing.

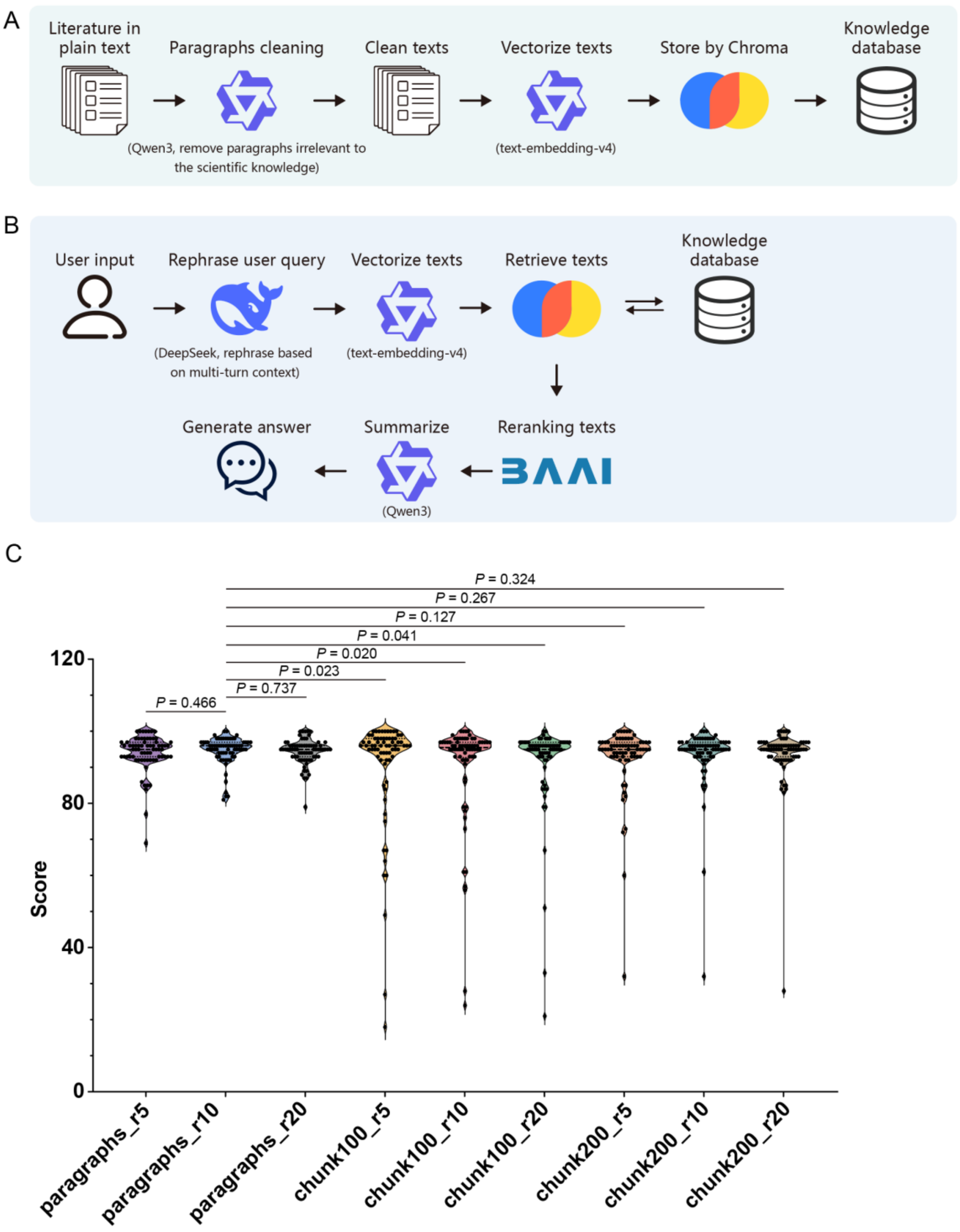

Fig. S4. Development of potato knowledge database.

(A) Pipeline of paper cleaning and vectorization.

(B) Pipeline of query and answer generation for potato knowledge.

(C) Scoring of generation results using different segmentation and fragment retrieval methods. 'Paragraphs', 'chunk100', and 'chunk200' represent segmentation by natural paragraphs, 100-word chunks with 60-word overlap, and 200-word chunks with 120-word overlap, respectively. 'r5', 'r10', and 'r20' represent the retrieval of the top 5, top

10, and top 20 fragments after reranking. Significance was assessed by Student's t-test.

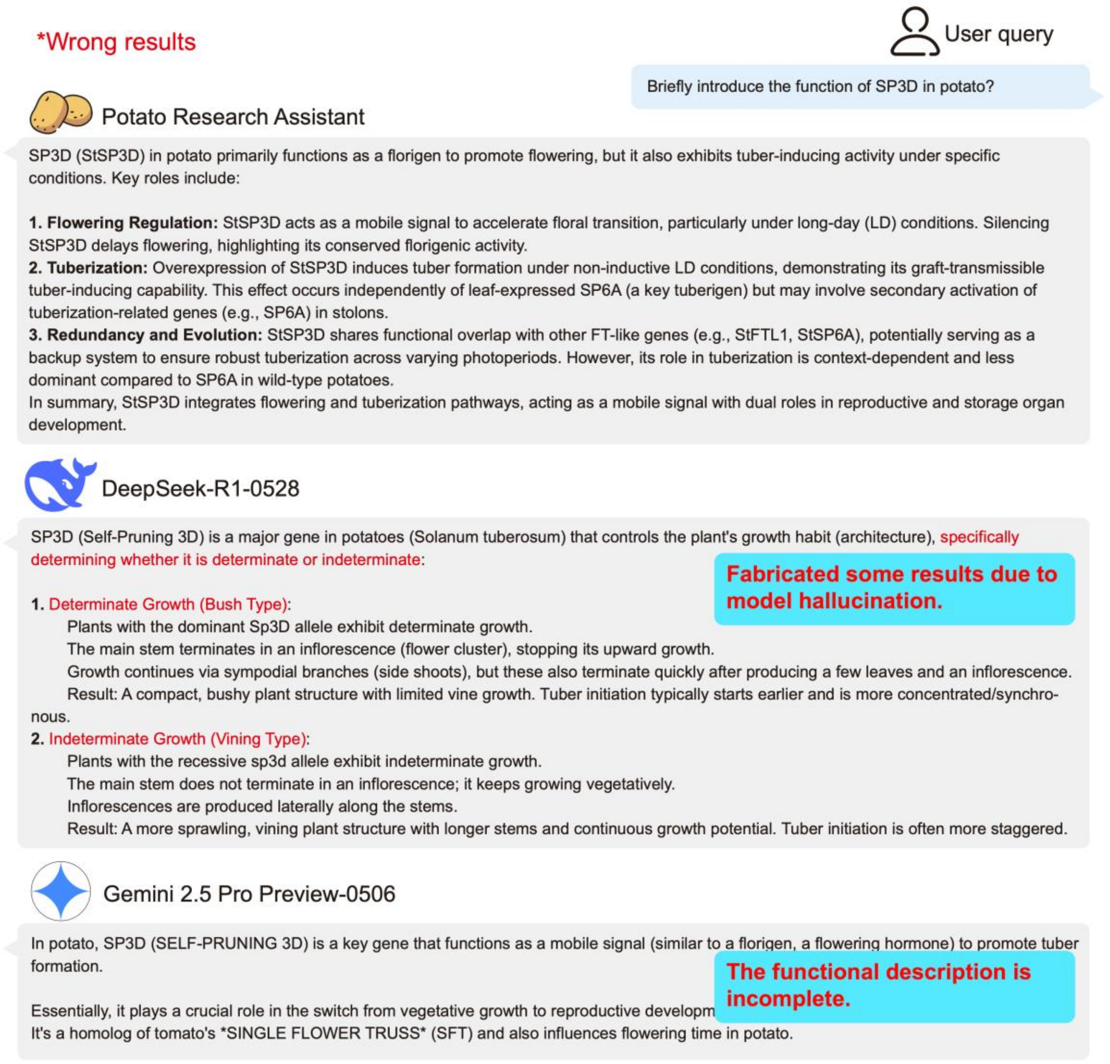

Fig. S5. Comparison of potato knowledge queries between Potato Research Assistant and general LLMs.

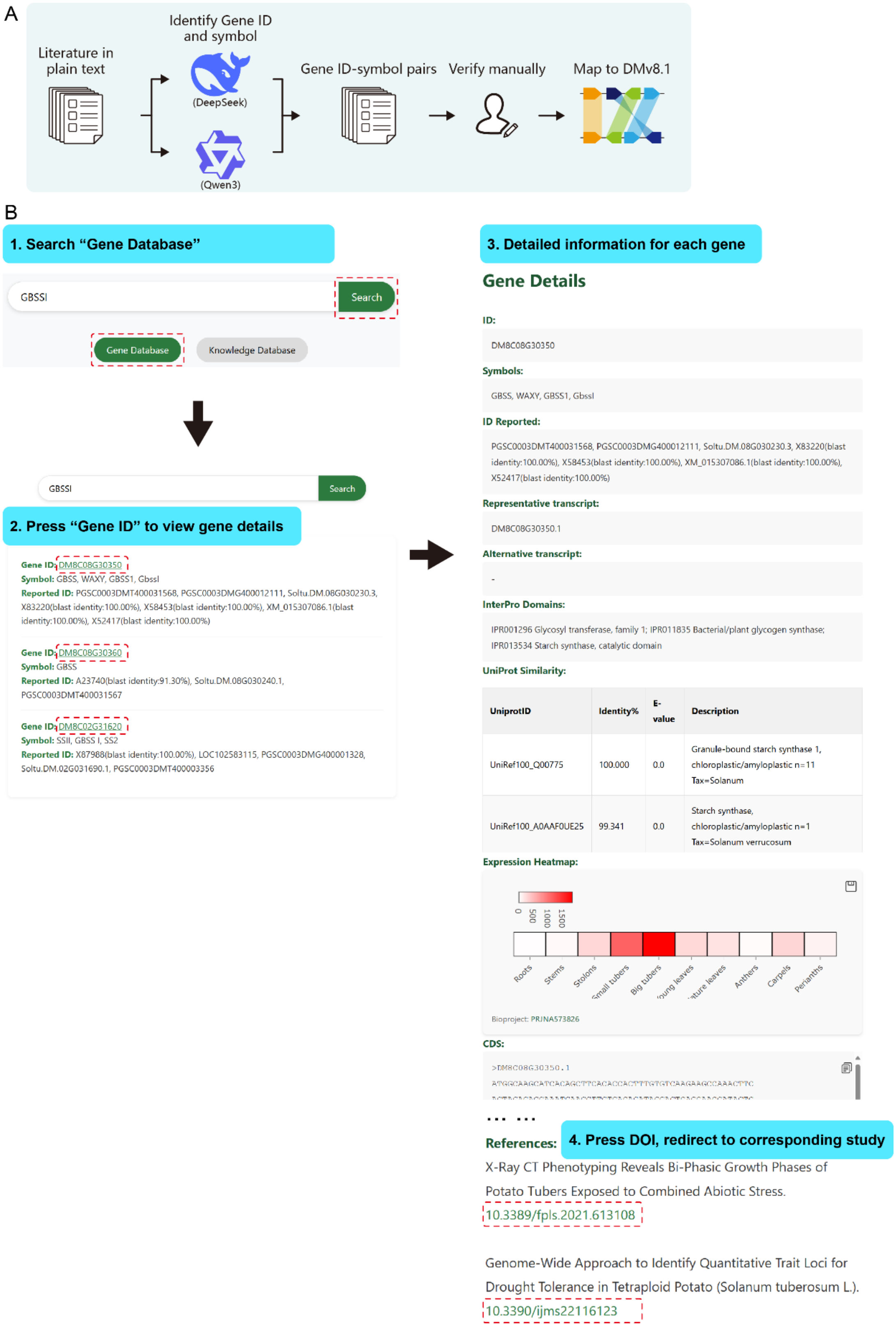

Fig. S6. Construction of potato gene database.

(A) Pipeline of reconciling gene IDs across historical studies.

(B) Function illustration of gene database.

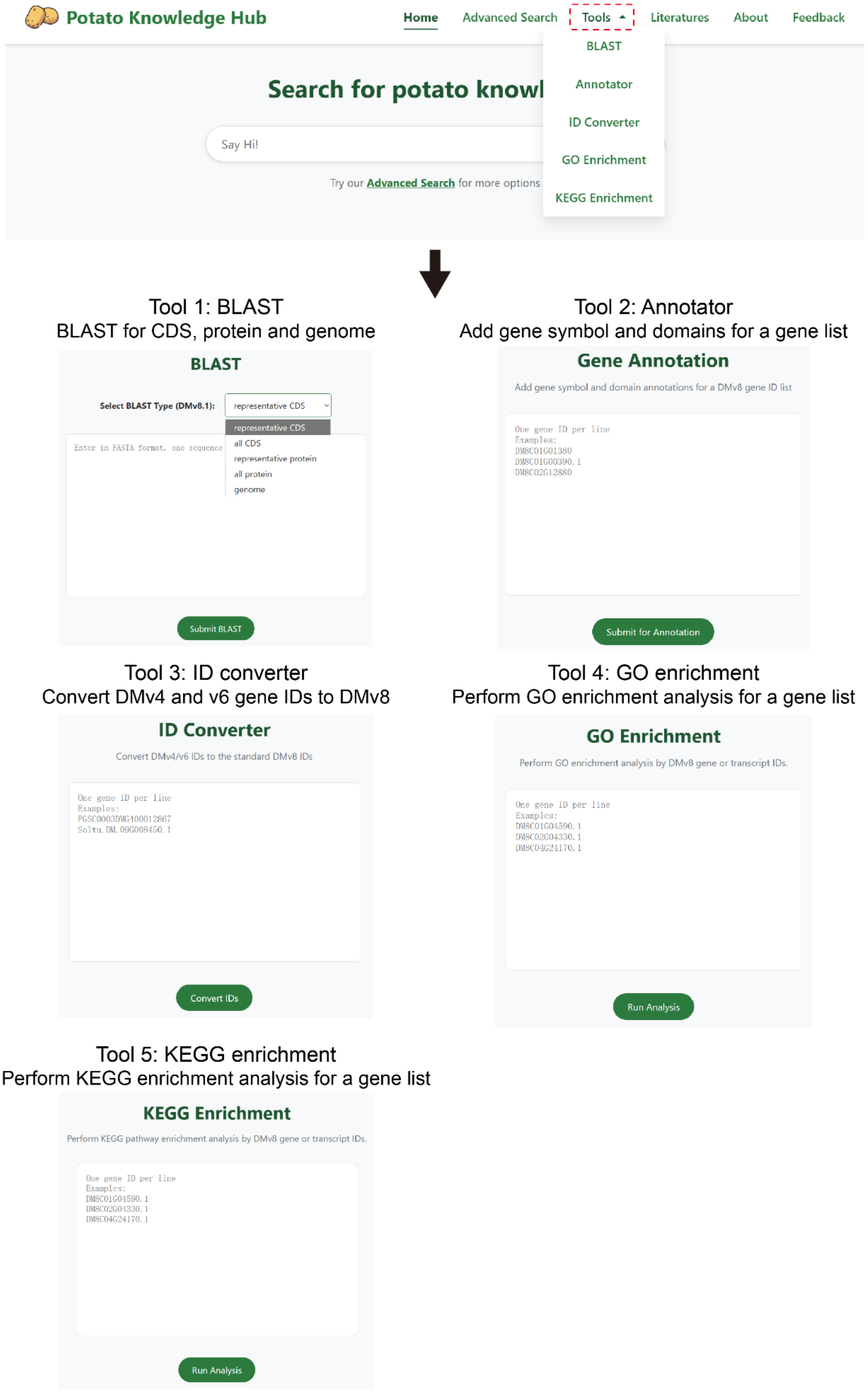

Fig. S7. Toolkit integrated in Potato Knowledge Hub.

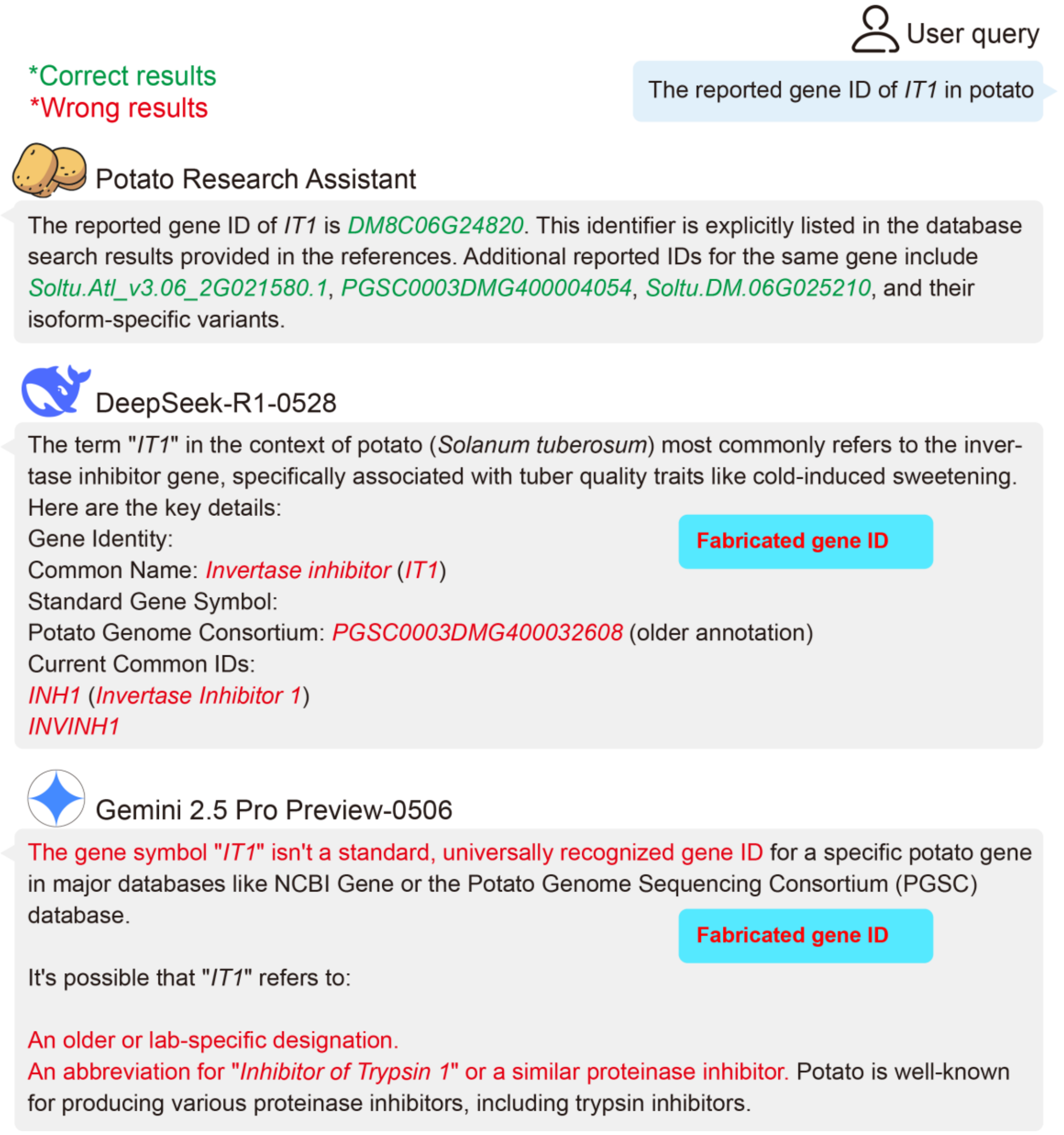

Fig. S8. Comparison of gene detail comprehension between potato research assistant and general LLMs.

The correct results were marked as green fonts, and the fabricated results were marked as red.

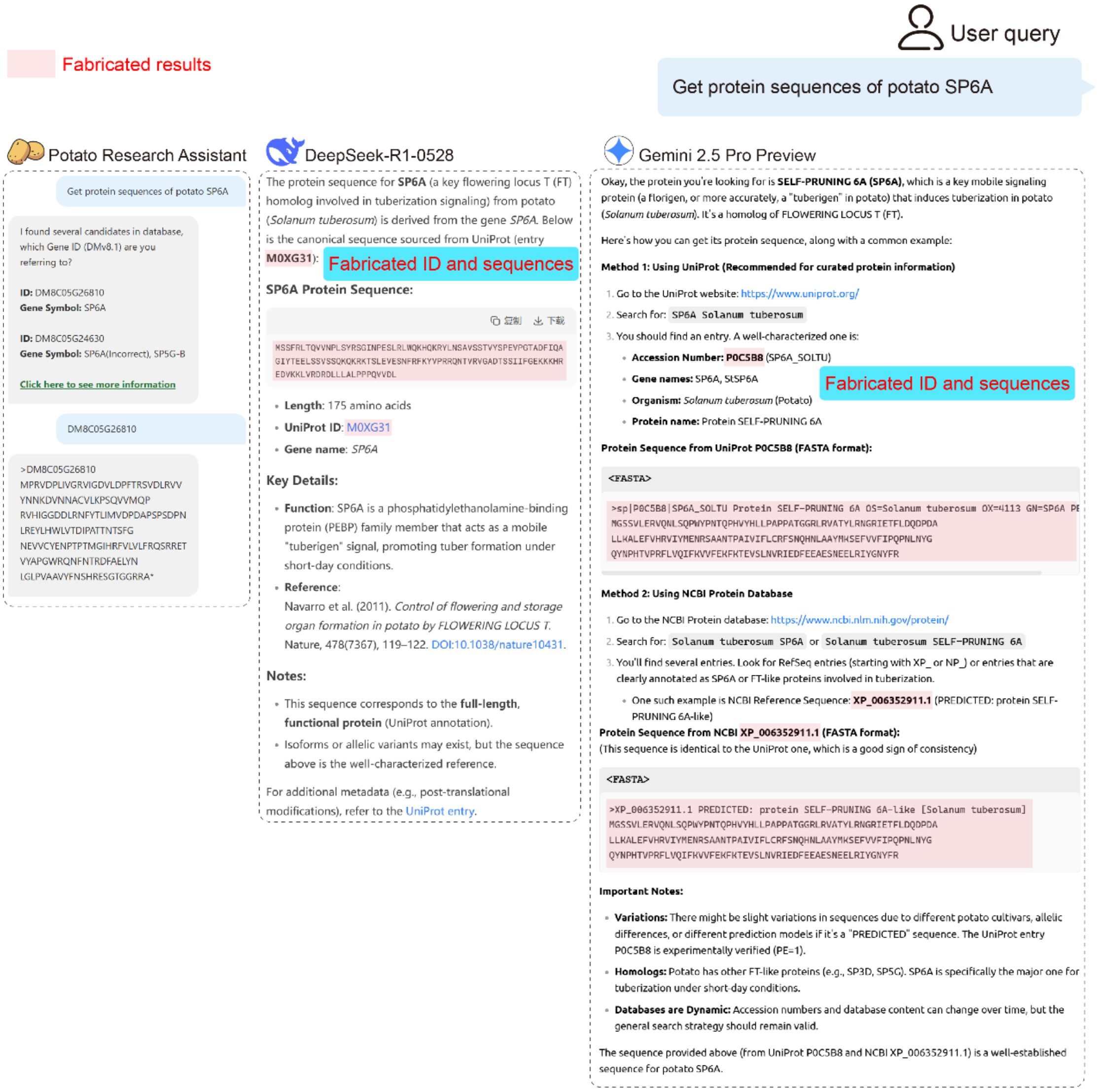

Fig. S9. Comparison of sequence extraction between potato research assistant and general LLMs.

The fabricated gene IDs and sequences generated by general LLMs were marked as pink background.

Supplementary References

Camacho, C., Coulouris, G., Avagyan, V., Ma, N., Papadopoulos, J., Bealer, K., and Madden, T.L. (2009). BLAST+: architecture and applications. BMC Bioinformatics **10**:421. 10.1186/1471-2105-10-421.

Chen, C., Wu, Y., Li, J., Wang, X., Zeng, Z., Xu, J., Liu, Y., Feng, J., Chen, H., He, Y., et al. (2023). TBtools-II: A "one for all, all for one" bioinformatics platform for biological big-data mining. Mol Plant **16**:1733-1742. 10.1016/j.molp.2023.09.010.

Chen, S. (2023). Ultrafast one-pass FASTQ data preprocessing, quality control, and deduplication using fastp. Imeta **2**:e107. 10.1002/imt2.107.

Jones, P., Binns, D., Chang, H.Y., Fraser, M., Li, W., McAnulla, C., McWilliam, H., Maslen, J., Mitchell, A., Nuka, G., et al. (2014). InterProScan 5: genome-scale protein function classification. Bioinformatics **30**:1236-1240. 10.1093/bioinformatics/btu031.

Kim, D., Langmead, B., and Salzberg, S.L. (2015). HISAT: a fast spliced aligner with low memory requirements. Nat Methods **12**:357-360. 10.1038/nmeth.3317.

Krumdick, M., Lovering, C., Reddy, V., Ebner, S., and Tanner, C. (2025). No free labels: Limitations of llm-as-a-judge without human grounding. arXiv preprint arXiv:2503.05061.

Pertea, M., Kim, D., Pertea, G.M., Leek, J.T., and Salzberg, S.L. (2016). Transcript-level expression analysis of RNA-seq experiments with HISAT, StringTie and Ballgown. Nat Protoc **11**:1650-1667. 10.1038/nprot.2016.095.

Suzek, B.E., Wang, Y., Huang, H., McGarvey, P.B., Wu, C.H., and UniProt, C. (2015). UniRef clusters: a comprehensive and scalable alternative for improving sequence similarity searches. Bioinformatics **31**:926-932. 10.1093/bioinformatics/btu739.

Szymanski, A., Ziems, N., Eicher-Miller, H.A., Li, T.J.-J., Jiang, M., and Metoyer, R.A. (2025). Limitations of the llm-as-a-judge approach for evaluating llm outputs in expert knowledge tasks. Proceedings of the 30th International Conference on Intelligent User Interfaces.

Tang, H., Krishnakumar, V., Zeng, X., Xu, Z., Taranto, A., Lomas, J.S., Zhang, Y., Huang, Y., Wang, Y., Yim, W.C., et al. (2024). JCVI: A versatile toolkit for comparative genomics analysis. Imeta **3**:e211. 10.1002/imt2.211.

Wang, B., Xu, C., Zhao, X., Ouyang, L., Wu, F., Zhao, Z., Xu, R., Liu, K., Qu, Y., Shang, F., et al. (2024). MinerU: An open-source solution for precise document content extraction. arXiv preprint

arXiv:2409.18839.

Wickham, H. (2016). ggplot2 : Elegant Graphics for Data Analysis. 2nd ed. Springer-Verlag New York.

Wu, T., Hu, E., Xu, S., Chen, M., Guo, P., Dai, Z., Feng, T., Zhou, L., Tang, W., Zhan, L., et al. (2021). clusterProfiler 4.0: A universal enrichment tool for interpreting omics data. Innovation (Camb) **2**:100141. 10.1016/j.xinn.2021.100141.
