# Supplementary material for: An AI-powered knowledge hub for potato functional genomics
Source: Plant Commun. 2026 Jan 14;7(5):101730. doi: 10.1016/j.xplc.2026.101730 (PMC13174205; doi:10.1016/j.xplc.2026.101730)
Supplement: Document S2. Article plus supplemental information [file mmc2.pdf]

Plant Communications
Correspondence

CellPress
Partner Journal

An AI-powered knowledge hub for potato functional genomics

Dear Editor,

Potato is one of the most important tuber crops, feeding approximately 1.3 billion people worldwide. Functional gene research is fundamental for enhancing crop yield, quality, and resilience to the rapidly changing global climate (Wang et al., 2025). Potato functional genomics has advanced significantly in recent years (Deng et al., 2024; Plunkert et al., 2024; Lian et al., 2025). However, iterative updates to the potato reference genome (DM1-3 516 R44) have introduced incompatible gene nomenclature systems, creating persistent challenges in reconciling gene IDs and symbols across historical studies (Supplemental Figure 1). In parallel, an ∼100% increase in related publications over the past decade has overwhelmed conventional literature mining methods (Supplemental Figure 2). Collectively, these issues are hindering progress in potato functional genomics. The recent emergence of large language models (LLMs) represents a paradigm-shifting advance in processing exabyte-scale heterogeneous data (Yang et al., 2025; Zhang et al., 2025). Subsequently, AI agent systems have been developed to extend LLMs' capabilities from cognitive assistance ("knowing how") to operational autonomy ("getting it done"), creating transformative opportunities for addressing data-intensive challenges across diverse biological research domains.

To leverage LLMs and AI agents for advancing potato research and to demonstrate their potential in functional genomics, we present the Potato Knowledge Hub (https://www.potato-ai.top; Figure 1), a freely accessible platform for the potato research community. This integrated platform comprises three key modules: (1) a potato knowledge database powered by a retrieval-augmented generation (RAG) architecture, drawing from over 6112 high-quality potato research articles, which overcomes key limitations of general LLMs (static knowledge and hallucination risk) to provide accurate scientific responses; (2) a manually curated functional gene database based on the latest potato genome assembly, featuring 2853 reported functional genes with cross-version ID mapping; and (3) a practical toolkit incorporating BLAST, ID conversion, gene annotation, and Gene Ontology/Kyoto Encyclopedia of Genes and Genomes enrichment analyses for efficient data processing. Moreover, we developed the Potato Research Assistant, an AI agent that integrates all platform modules to facilitate intuitive, natural language-based interaction. The Potato Knowledge Hub is designed to provide a streamlined user experience, freeing researchers from labor-intensive literature mining and fragmented analytical workflows. By substantially enhancing individual research efficiency, this platform promises to accelerate discoveries across the field of potato functional genomics.

The first step in developing the Potato Knowledge Hub was to construct a high-quality, specialized potato research corpus. To comprehensively capture potato research, we searched NCBI PubMed and the Web of Science Core Collection using "potato" as a keyword in titles or abstracts. This search, spanning from January 1, 1900, to the present, retrieved over 57 795 nonredundant papers (Supplemental Figure 2). To enrich for high-quality studies, we consulted the Chinese Academy of Sciences journal ranking and extracted research articles published in two top-tier journal categories (Q1 and Q2) in biology, agriculture, and forestry sciences and multidisciplinary categories, yielding 21 505 papers. We then implemented LLMs to analyze titles and abstracts and to exclude studies that only mentioned "potato" without using potato as the research subject. This screening yielded 6007 studies relevant to potato breeding, genetics, metabolism, physiology, genomics, and functional gene research. We further manually reviewed report- or letter-type papers lacking abstract information and retrieved 105 more papers. To assess database coverage, we manually reviewed the 100 most recent publications in potato functional gene research and confirmed that all were included. In total, we collected 6112 high-quality potato research papers. To ensure detailed and accurate content, we downloaded full-text Portable Document Formats, rather than relying only on abstracts. Each Portable Document Format was converted to plain text for downstream data processing (Supplemental Figure 3).

Paragraphs from each paper were analyzed using an LLM to exclude nonscientific sections, including author information, affiliations, references, and acknowledgments. A RAG architecture was applied to enhance response accuracy and reduce hallucinations. The full text of each paper was segmented and vectorized using an embedding model. To support multi-turn interactions, an LLM analyzed the user query in its conversational context and rephrased it to improve knowledge retrieval efficiency. The rephrased query was vectorized using the same model, and the top 200 relevant paragraphs were retrieved based on embedding distance. A re-ranking model then rescored semantic relevance between each segment and the query. The highest-ranking segments, together with the user query, were submitted to the LLM to generate the final answer (Supplemental Figure 4A and 4B).

To determine the optimal chunking and retrieval settings that maximize performance, we evaluated three chunking methods (100 words with 60-word overlap, 200 words with 120-word overlap, and paragraph-based chunking) combined with three retrieval depths (top 5, 10, and 20 chunks after re-ranking). We collected 70 potato knowledge questions and corresponding validated answers provided by professors, postdoctoral researchers, and graduate students. We then employed an "LLM as a judge" framework, which provides a scalable and interpretable proxy for human evaluation (Zheng et al., 2023). Specifically, a third-party LLM (GPT-o3) acted as the judge and scored

 © 2026 The Author(s). Published by Elsevier Inc. on behalf of CAS Center for Excellence in Molecular Plant Sciences, Chinese Academy of Sciences, and Chinese Society for Plant Biology.
This is an open access article under the CC BY license (http://creativecommons.org/licenses/by/4.0/).

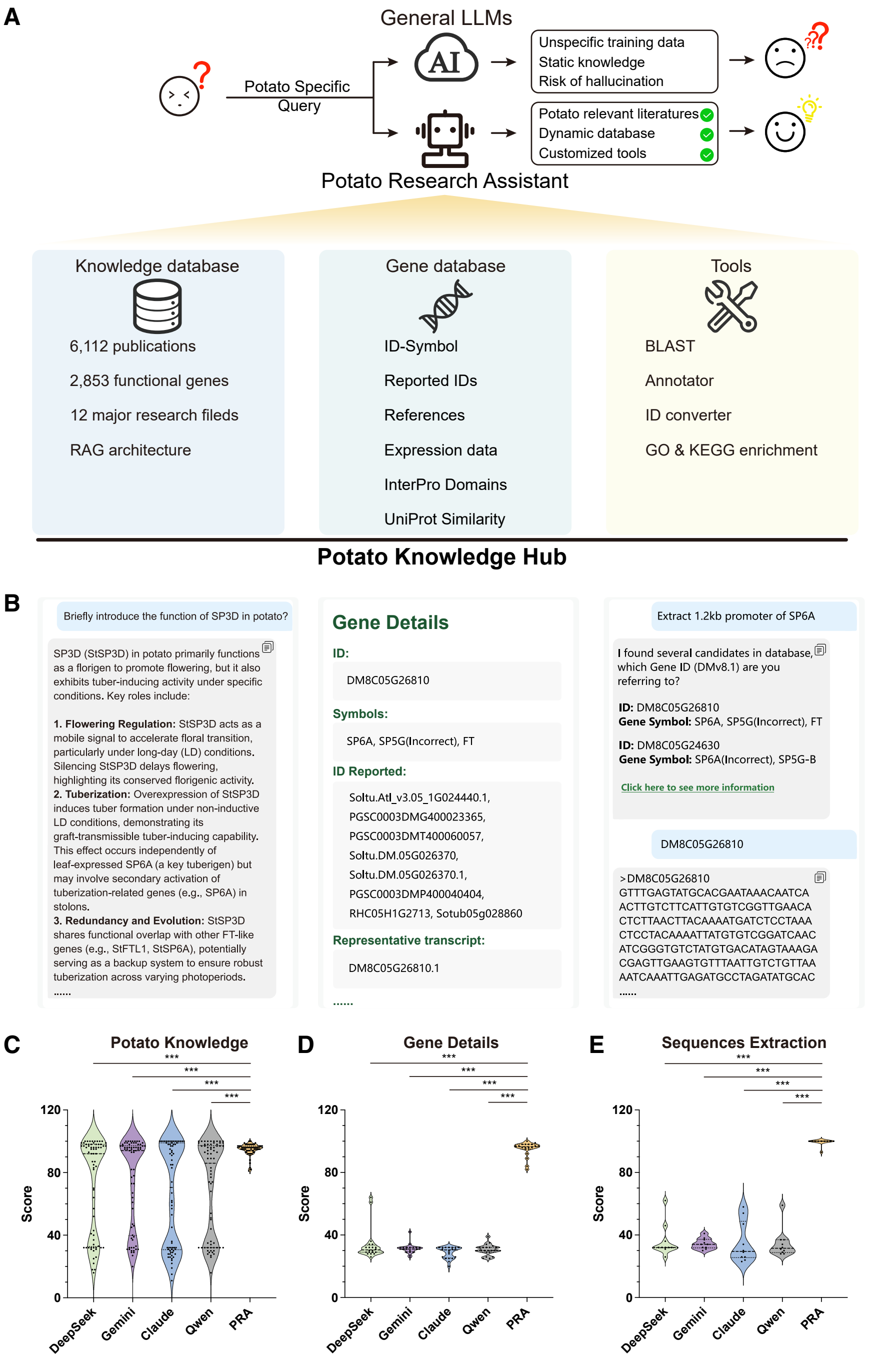

Figure 1. Architecture and performance evaluation of the Potato Knowledge Hub.

(A) Schematic representation of the Potato Knowledge Hub system architecture. RAG, retrieval-augmented generation.

(B) Overview of the functional modules of the Potato Knowledge Hub.

(C–E) Performance comparison between the Potato Research Assistant (PRA) and general LLMs in **(C)** potato knowledge queries, **(D)** gene detail comprehension, and **(E)** sequence extraction. Statistical significance was assessed using Student's *t*-test. ***$P < 0.001$.

responses against the validated answers across four dimensions: knowledge accuracy (40 points), completeness (30 points), relevance (20 points), and clarity (10 points).

The results indicate that paragraph-based segmentation yields higher and more concentrated scores. Conversely, fixed-length segmentation exhibits a noticeably dispersed score distribution across all retrieval depths even though the evaluation scores obtained with 200-word segmentation did not differ significantly from those of paragraph-based segmentation. Under paragraph-based segmentation, retrieving the top 5 segments led to incomplete answers for some questions because of insufficient reference content, resulting in scores below 70. The score distributions obtained when retrieving the top 10 or top 20 natural paragraphs were highly similar (Supplemental Figure 4C). Consequently, we adopted paragraph-based text segmentation and retrieval of the top 10 segments for LLM-based answer generation. This curated potato research corpus, combined with the RAG-based framework, enables precise, domain-specific answers with minimal hallucination risk (Figure 1B and 1C and Supplemental Figure 5).

The continuously updated potato reference genomes, together with incompatible gene nomenclature systems, pose a persistent challenge for reconciling gene IDs and symbols across historical studies (Potato Genome Sequencing et al., 2011; Pham et al., 2020; Yang et al., 2023) (Supplemental Figure 1). To address this issue, we developed an LLM-driven gene entity normalization workflow to accurately map previously reported genes to the latest genome assembly (DMv8.1). An LLM was employed to analyze the full plain text of each paper and extract reported functional gene symbols and corresponding IDs. Two independent rounds of extraction were performed using different LLMs to maximize coverage. The merged results were then subjected to manual curation, yielding 4947 gene ID–symbol pairs. These gene IDs originated from multiple databases and genome versions, including GenBank, UniProt, Solanaceae Genomics Network, ATL_v3, SolTub3.0, DMv4.03, DMv6.1, and DMv8.1. All identifiers were mapped to the DMv8.1 reference genome using genome synteny and BLAST-based sequence identity. This process resulted in a final set of 2853 non-redundant functional genes (Supplemental Figure 6A). During manual curation, we also identified and corrected historical nomenclature errors (for example, SP5G-B being mislabeled as SP6A; Herrera-Isidron et al., 2024), with all corrections annotated and traceable in our database. To ensure full transparency, our database maintains direct links connecting DMv8.1 gene IDs, legacy identifiers, and

gene symbols associated with their respective source publications. To further enhance research utility, we integrated key functional annotations, including InterPro domains, UniProt similarity information, and expression patterns, into each gene entry (Figure 1B and Supplemental Figure 6B). We also developed a toolkit comprising BLAST, Annotator, ID converter, and Gene Ontology and Kyoto Encyclopedia of Genes and Genomes enrichment tools to facilitate rapid BLAST queries, gene domain and symbol annotation, DMv4 and DMv6 to DMv8 ID conversion, and functional enrichment analyses (Supplemental Figure 7). Together, this gene database enables researchers to retrieve comprehensive gene information and associated literature using either current gene symbols or legacy identifiers, significantly simplifying the background research process for functional gene studies and facilitating efficient project initiation.

To optimize user interaction with the Potato Knowledge Hub, we implemented an agentic task orchestration architecture and developed the Potato Research Assistant, an AI agent capable of invoking the workflows of the knowledge database, gene database, and integrated toolkit (Figure 1A). This agent interacts with users via natural language, conducts intent analysis, and calls the appropriate internal scripts to summarize potato research knowledge, retrieve gene information and sequences, and execute specific analytical tools. For a queried gene symbol, the assistant can summarize historical research, extract sequences (coding, protein, and customized promoter regions, etc.), and provide detailed information on putative gene function. By integrating these capabilities, the AI agent liberates users from tedious literature mining and complex bioinformatics software, enabling scientists without specialized computational expertise to efficiently explore and utilize a tremendous amount of gene information with ease.

To evaluate the performance of the Potato Research Assistant, in addition to the 70 questions used to benchmark the knowledge base, we collected an additional 20 questions focused on gene understanding and 10 questions related to sequence extraction to assess the gene database. We compared the responses generated by the Potato Research Assistant with those produced by state-of-the-art general LLMs; specifically, the open-source models DeepSeek-R1-0528 and Qwen3-235B-A22B (thinking enabled) as well as the closed-source models Gemini 2.5 Pro Preview-0506 and Claude Sonnet 4. Responses from both the Potato Research Assistant and the general LLMs were evaluated using the same scoring system applied in the previous RAG evaluation. The Potato Research Assistant outperformed the general LLMs across all three evaluation categories. This performance advantage underscores the effectiveness of our agentic task orchestration architecture and specialized database design in enabling superior domain expert knowledge mastery compared with general-purpose LLMs. For knowledge retrieval tasks, the assistant showed higher performance stability, whereas general LLMs exhibited score polarization, likely due to an incomplete training corpus (Figure 1C and Supplemental Figure 5). For gene detail comprehension and sequence extraction, the assistant demonstrated clear advantages, supported by its dedicated potato gene database (Figure 1D and 1E and Supplemental Figures 8 and 9).

Our work addresses key challenges in potato functional genomics research, which is representative of crops with high genetic complexity and frequent genome version inconsistencies. The Potato Knowledge Hub will be actively updated on a monthly basis to integrate the latest advances in potato research. We anticipate that this hub will serve as a valuable community resource for advancing potato functional genomics and providing crucial information to support potato breeding. Furthermore, the technical strategies developed here, including the LLM-driven gene entity normalization workflow and the agentic task orchestration architecture, offer a transferable framework for developing similar platforms in other areas of biological research.

DATA AND CODE AVAILABILITY

Transcriptome data were obtained from NCBI (BioProject: PRJNA573826). The code for the Potato Knowledge Hub is hosted on Gitee (https://gitee.com/yuxin_2023/potato-knowledge-hub). Benchmark questions, manually curated reference answers, model responses, and evaluation criteria are available on Figshare (https://doi.org/10.6084/m9.figshare.29815859). The UniRef100 database was downloaded from the UniProt FTP server (https://ftp.uniprot.org/).

FUNDING

This work was supported by the Guangdong Major Project of Basic and Applied Basic Research (2021B0301030004), the National Natural Science Foundation of China (32488302, 32302582, and 32402608), the Agricultural Science and Technology Innovation Program (CAAS-ZDRW202404), the Yunnan Fundamental Research Projects (grants 202501BC070003 and 202501AT070005), the China Postdoctoral Science Foundation (BX20200376), and the Yunnan Province Xingdian Talent Support Program.

ACKNOWLEDGMENTS

We acknowledge technical support from the Intelligent Computing Center of Yunnan Normal University. We thank Shuhua Yang (China Agricultural University) and Dawei Li, Pei Wang, and Pingxian Zhang (Chinese Academy of Agricultural Sciences) for valuable suggestions that improved the Potato Knowledge Hub. We also thank all researchers who participated in platform testing for performance and stability and who provided constructive feedback. No conflict of interest is declared.

AUTHOR CONTRIBUTIONS

S.H., Y.Z., and Yuxin Jia conceived the study. Yuxin Jia and Y.Z. wrote the manuscript. Yudong Jia, Yuxin Jia, and J. Li developed the code for the Potato Knowledge Hub. J. Li performed the bioinformatics analyses. Yuxin Jia, Y.Z., J. Li, F.L., X.S., J. Luo, Y.D., C.S., Q.C., L.W., and A.L. collected literature, manually curated gene IDs and symbols, and tested the functionality of the Potato Knowledge Hub. S.H., Y.S., Yuxin Jia, and Y.Z. discussed and revised the manuscript. All authors read and approved the final version.

SUPPLEMENTAL INFORMATION

Supplemental information is available at *Plant Communications Online*.

Received: August 10, 2025
Revised: December 16, 2025
Accepted: January 9, 2026
Published: January 14, 2026

Jinye Li[1,3], Yudong Jia[4], Futing Li[1,3], Xiaoqi Su[1,3], Jilin Luo[1,3], Yarui Dong[1], Chunyan Sun[1,3], Qinghan Cui[1,3], Li Wang[1], Axiu Li[1,3], Yi Shang[1,3], Sanwen Huang[2], Yujuan Zhu[2,] and Yuxin Jia[1,3,*]*

[1]Engineering Research Center for Valorization of Unique Bio-Resources in Yunnan, Ministry of Education, School of Life Sciences, Yunnan Normal University, Kunming 650500, China
[2]Shenzhen Branch, Guangdong Laboratory of Lingnan Modern Agriculture, Genome Analysis Laboratory of the Ministry of Agriculture and Rural Affairs, Agricultural Genomics Institute at Shenzhen, Chinese Academy of Agricultural Sciences, Shenzhen, Guangdong 518120, China
[3]Southwest United Graduate School, Kunming 650092, China
[4]School of Information and Communication Engineering, Beijing University of Posts and Telecommunications, Beijing 100876, China
***Correspondence: Yujuan Zhu (zhuyujuan@caas.cn), Yuxin Jia (jiayuxin@ynnu.edu.cn)**
https://doi.org/10.1016/j.xplc.2026.101730

REFERENCES

Deng, R., Huang, S., Du, J., Luo, D., Liu, J., Zhao, Y., Zheng, C., Lei, T., Li, Q., Zhang, S., et al. (2024). The brassinosteroid receptor StBRI1 promotes tuber development by enhancing plasma membrane H +-ATPase activity in potato. Plant Cell **36**:3498–3520. https://doi.org/10.1093/plcell/koae163.

Herrera-Isidron, L., Valencia-Lozano, E., Uribe-Lopez, B., Delano-Frier, J.P., Barraza, A., and Cabrera-Ponce, J.L. (2024). Molecular Insights into the Role of Sterols in Microtuber Development of Potato Solanum tuberosum L. Plants **13**:2391. https://doi.org/10.3390/plants13172391.

Lian, Q., Zhang, Y., Zhang, J., Peng, Z., Wang, W., Du, M., Li, H., Zhang, X., Cheng, L., Du, R., et al. (2025). A genomic variation map provides insights into potato evolution and key agronomic traits. Mol. Plant **18**:570–589. https://doi.org/10.1016/j.molp.2025.01.016.

Pham, G.M., Hamilton, J.P., Wood, J.C., Burke, J.T., Zhao, H., Vaillancourt, B., Ou, S., Jiang, J., and Buell, C.R. (2020). Construction of a chromosome-scale long-read reference genome assembly for potato. GigaScience **9**:giaa100. https://doi.org/10.1093/gigascience/giaa100.

Plunkert, M.L., Martínez-Gómez, J., Madrigal, Y., Hernández, A.I., and Tribble, C.M. (2024). Tuber, or not tuber: Molecular and morphological basis of underground storage organ development. Curr. Opin. Plant Biol. **80**:102544. https://doi.org/10.1016/j.pbi.2024.102544.

Potato Genome Sequencing Consortium, Xu, X., Pan, S., Cheng, S., Zhang, B., Mu, D., Ni, P., Zhang, G., Yang, S., Li, R., et al. (2011). Genome sequence and analysis of the tuber crop potato. Nature **475**:189–195. https://doi.org/10.1038/nature10158.

Wang, N., Li, H., and Huang, S. (2025). Rational Redomestication for Future Agriculture. Annu. Rev. Plant Biol. **76**:637–662. https://doi.org/10.1146/annurev-arplant-083123-064726.

Yang, F., Kong, H., Ying, J., Chen, Z., Luo, T., Jiang, W., Yuan, Z., Wang, Z., Ma, Z., Wang, S., et al. (2025). SeedLLM.Rice: A large language model integrated with rice biological knowledge graph. Mol. Plant **18**:1118–1129. https://doi.org/10.1016/j.molp.2025.05.013.

Yang, X., Zhang, L., Guo, X., Xu, J., Zhang, K., Yang, Y., Yang, Y., Jian, Y., Dong, D., Huang, S., et al. (2023). The gap-free potato genome assembly reveals large tandem gene clusters of agronomical importance in highly repeated genomic regions. Mol. Plant **16**:314–317. https://doi.org/10.1016/j.molp.2022.12.010.

Zhang, R., Wang, Y., Yang, W., Wen, J., Liu, W., Zhi, S., Li, G., Chai, N., Huang, J., Xie, Y., et al. (2025). PlantGPT: An Arabidopsis-Based Intelligent Agent that Answers Questions about Plant Functional Genomics. Adv. Sci. **1212**:e03926. https://doi.org/10.1002/advs.202503926.

Zheng, L.M., Chiang, W.L., Sheng, Y., Zhuang, S.Y., Wu, Z.H., Zhuang, Y.H., Lin, Z., Li, Z.H., Li, D.C., Xing, E.P., et al. (2023). Judging LLM-as-a-Judge with MT-Bench and Chatbot Arena. Adv. Neural Inf. Process. Syst. **36**:46595–46623.

Plant Communications, Volume 7

Supplemental information

An AI-powered knowledge hub for potato functional genomics

Jinye Li, Yudong Jia, Futing Li, Xiaoqi Su, Jilin Luo, Yarui Dong, Chunyan Sun, Qinghan Cui, Li Wang, Axiu Li, Yi Shang, Sanwen Huang, Yujuan Zhu, and Yuxin Jia

Supplementary Information

Title: An AI-powered Knowledge Hub for Potato Functional Genomics

Authors:

Jinye Li[1,3], Yudong Jia[4], Futing Li[1,3], Xiaoqi Su[1,3], Jilin Luo[1,3], Yarui Dong[1], Chunyan Sun[1,3], Qinghan Cui[1,3], Li Wang[1], Axiu Li[1,3], Yi Shang[1,3], Sanwen Huang[2], Yujuan Zhu[2,*], Yuxin Jia[1,3,*]

Affiliations

[1]Engineering Research Center for Valorization of Unique Bio-Resources in Yunnan, Ministry of Education, School of Life Sciences, Yunnan Normal University, Kunming, 650500, China.

[2]Shenzhen Branch, Guangdong Laboratory of Lingnan Modern Agriculture, Genome Analysis Laboratory of the Ministry of Agriculture and Rural Affairs, Agricultural Genomics Institute at Shenzhen, Chinese Academy of Agricultural Sciences, Shenzhen, Guangdong 518120, China.

[3]Southwest United Graduate School, Kunming 650092, China.

[4]School of Information and Communication Engineering, Beijing University of Posts and Telecommunications, Beijing, 100876, China.

[*]Correspondence to: jiayuxin@ynnu.edu.cn (Y.J.), zhuyujuan@caas.cn (Y.Z.)

Contents

METHODS

Fig. S1. Examples of inconsistent gene nomenclature systems across different versions of the potato reference genome.

Fig. S2. The number of publications on potato research has increased significantly in recent years.

Fig. S3. Pipeline of literature processing.

Fig. S4. Development of potato knowledge database.

Fig. S5. Comparison of potato knowledge queries between Potato Research

Assistant and general LLMs.

Fig. S6. Construction of potato gene database.

Fig. S7. Toolkit integrated in Potato Knowledge Hub.

Fig. S8. Comparison of gene detail comprehension between Potato Research Assistant and general LLMs.

Fig. S9. Comparison of sequence extraction between Potato Research Assistant and general LLMs.

METHODS

Processing literatures

Potato-related literature was retrieved by searching the titles or abstracts from the Web of Science Core Collection and PubMed, with "Potato" as the keyword. Relevant studies, including their titles, abstracts, journal information, and DOIs, were exported for subsequent filtering. Papers published in Q2 or higher-ranked journals, according to the CAS Journal Ranking (2023), were retained as high-quality literature. Furthermore, the title and abstract of each paper were then submitted to a LLM to screen out papers that merely mentioned "potato" in the title or abstract but were not focused on potato research fields. The screening was performed twice using DeepSeek-R1-0528 (671B) and Qwen3-235B-A22B (enable thinking), separately. To retain studies more comprehensively, we kept studies that were present in the results from either screen. Literature lacking abstract information underwent further manual review. This collection was designated as high-quality potato literature. Full PDFs of these selected studies were downloaded, and their plain text was extracted using MinerU (v1.2.2), preserving the original texts organization (Wang et al., 2024).

Potato literature knowledge database construction

To improve the quality of data retrieval, we performed data cleaning to remove the text that was not related to the scientific content in the literature. We submitted each paragraph of an article to Qwen3-235B-A22B to filter out authors, affiliations, references, acknowledgements, standalone section titles, page headers, footers, as well as formulas. The vectorization model "text-embedding-v4", developed by the Qwen3 group, was employed to vectorize the collected literature into 2048 dimensions. Each paragraph was treated as an individual chunk for this process. The vectorized data, original text and DOI were stored in Chroma database (1.0.13, https://github.com/chroma-core/chroma).

For data retrieval, the DeepSeek-V3-250324 model was used to rephrase and optimize user's query for vector retrieval, for example, understand user intent based on

multi-turn conversation contexts, add abbreviations to full form phrases or expand abbreviations. The query was vectorized using the same parameters and used to search the Chroma database using the query() function. The top 200 chunks exhibiting the highest similarity scores were extracted. To further identify the most relevant chunks, the reranking model BGE-reranker-v2-m3 was utilized to assess the relevance between these retrieved chunks and the query. The user's query, along with the top relevant chunks, was then sent to the Qwen3-235B-A22B (enable thinking) for summarization.

Potato gene database construction

To extract gene IDs and their corresponding symbols, the plain text of each paper was submitted to DeepSeek-R1-0528 (671B) and Qwen3-235B-A22B (enable thinking) for analysis separately. The resulting data were subsequently merged. All gene ID and symbol correspondences were then manually verified to correct potential inaccuracies. To anchor reported gene IDs to the DMv8.1 reference genome, pairwise synteny analyses were conducted between DMv8.1 and other potato genomes (RH89-039-16, ATL_v3, Jan v1.1, SolTub3.0, DMv4.03, and DMv6.1) using MCscan within the JCVI toolkit (v1.3.6) to identify orthologs (Tang et al., 2024). For genes where syntenic orthologs could not be identified, and for gene sequences sourced from the Sol Genomics Network, UniProt, NCBI GenBank, and the Agria genome, orthologs in DMv8.1 were identified using the NCBI BLAST+ toolkit (v2.16.0) with default parameters and a sequence identity threshold of >90%. Finally, gene IDs, symbols, reported IDs, and their corresponding reference papers were compiled into an SQLite database for efficient data querying.

InterPro domain annotation, uniport similarity and expression analysis of each gene

For comprehensive functional domain annotation and expression analysis of protein-coding genes in the DMv8.1 genome, we performed the analysis in the following steps. First, protein sequences (with stop codons removed) were analyzed using InterProScan (v5.74-105.0) to identify functional domains, and the corresponding InterPro (IPR)

identifiers and domain descriptions were extracted (Jones et al., 2014). In parallel, homology searches were conducted using blastp (v2.14.0+) against the UniRef100 database, retaining only high-confidence matches (E-value < 1×10^{-10}) and selecting the top five hits with the highest sequence identity for functional inference (Suzek et al., 2015). For transcriptomic profiling, publicly available potato RNA-seq datasets were retrieved from the NCBI SRA database (PRJNA573826). Raw sequencing reads were processed for quality control using fastp (v0.24.0), then mapped to the DMv8.1 reference genome using HISAT2 (v2.1.0). Transcript abundance was quantified using StringTie (v2.2.3) and normalized as Transcripts Per Million (TPM) (Chen, 2023; Kim et al., 2015; Pertea et al., 2016). For samples with biological replicates, the mean TPM value across replicates was used to represent the final expression level of each gene, ensuring robust quantification.

AI agent and Potato knowledge hub construction

The "Potato Research Assistant" agent is powered by the Doubao-seed-1.6 LLM (with thinking enabled), which was accessed directly via its API (https://www.volcengine.com/). We employed the model as a frozen reasoning engine without conducting any further continuous pre-training or fine-tuning. The model acts as a reasoning engine designed to analyze user intent via natural language understanding, invoke external databases or tools for information retrieval, and synthesize the final answer based on the context. The Potato Knowledge Hub was developed using the Flask framework (v3.1.0, https://flask.palletsprojects.com/). Python was used to write the backend logical scripts, while HTML and JavaScript were employed for developing the web-based user interface.

API usage and research costs

The APIs for the DeepSeek and Doubao models were provided by VolcanoArk (https://www.volcengine.com/), while the Qwen3 model and the vectorization model text-embedding-v4 were supported by Alibaba Cloud (https://www.aliyun.com/). The API for the re-ranking model BGE-reranker-v2-m3 was provided by SiliconFlow

(https://cloud.siliconflow.cn/me/models). For research costs, constructing the literature knowledge base and functional gene database comprising 6,112 papers consumed approximately 50.1 M tokens for literature screening, 107.6 M tokens for text vectorization, and 2.0 G tokens for extracting gene ID and symbol correspondences.

Tools development

The BLAST tool was developed based on the NCBI BLAST+ toolkit (v2.16.0) (Camacho et al., 2009). The Annotator and ID converter were built using local Python scripts. Gene Ontology (GO) annotations were retrieved in OBO format from the OBO Foundry (http://purl.obolibrary.org/obo/go/go-basic.obo), while KEGG pathway annotations were extracted from the built-in TBtools.KeggBackEnd database in TBtools-II (Chen et al., 2023). The gene annotation file for the DMv8.1 genome was generated using the online platform eggNOG-mapper (http://eggnog-mapper.embl.de/) with default parameters. After establishing the correspondence between the annotation file and the background files, functional enrichment analysis was performed using the enricher function from the R package ClusterProfiler (v4.12.6), with the significance threshold set to pvalueCutoff = 0.05 and adjusted using the Benjamini–Hochberg correction (Wu et al., 2021). The enrichment results were visualized using the ggplot2 package (v3.5.2) (Wickham, 2016).

Performance evaluation

One hundred questions covering potato knowledge queries (70), gene detail comprehension (20), and sequence extraction (10) were collected from professors, postdocs, and graduate students, and the corresponding reference answers were generated and curated manually. We defined a 100-point assessment system consisting of four dimensions: knowledge accuracy (maximum 40 points), completeness (maximum 30 points), relevance (maximum 20 points) and clarity (maximum 10 points). We also carefully defined the judgment rules for each dimension. The LLM-as-a-judge framework was developed based on the reasoning model GPT-o3 (with reasoning_effort set to “high”). The questions, verified answers, meticulous judgment

rules, as well as the corresponding answers from the LLMs and Potato Research Assistant were submitted to GPT-o3 for scoring. Although the LLM-as-a-Judge framework offers scalability, we acknowledge that LLMs may not be entirely impartial and can be susceptible to biases inherent in their training or prompting (Krumdick et al., 2025; Szymanski et al., 2025). To mitigate these potential limitations, we utilized strictly defined judgment rules and expert-validated standard answers as the ground truth for all evaluations. The complete benchmark dataset—benchmark questions, manually curated standard answers, responses from both the Potato Research Assistant and general LLMs, judgment criteria, individual scores, and brief comments explaining each response and score—has been deposited in Figshare (https://doi.org/10.6084/m9.figshare.29815859).

Comparison of performance across different chunking strategies and retrieval counts

To evaluate the impact of different chunking strategies and retrieval depth on LLM generation performance, we compared three text segmentation granularities: fixed window chunking with 100 words/60-word overlap, 200 words/120-word overlap, and paragraph-based chunking. These were cross-tested with three retrieval depth after reranking (Top 5, 10, and 20), resulting in a total of nine test combinations. We selected 70 potato knowledge queries from the above benchmark dataset and employed the "LLM-as-a-judge" framework to automatically score the generation results for each combination. Detailed scoring data have been deposited in Figshare (https://doi.org/10.6084/m9.figshare.29815859).

SolTub_3.0	PGSC DM v4.03	DM v6.1	DM v8.1	Gene Symbol
NM_001287968.1	PGSC0003DMG400023365	Soltu.DM.05G026370	DM8C05G26810	SP6A
LOC102603307	PGSC0003DMG400018408	Soltu.DM.05G005140	DM8C05G04930	CDF1
NM_001288693.1	PGSC0003DMG400004054	Soltu.DM.06G025210	DM8C06G24820	IT1/BRC1b

Fig. S1. Examples of inconsistent gene nomenclature systems across different versions of the potato reference genome.

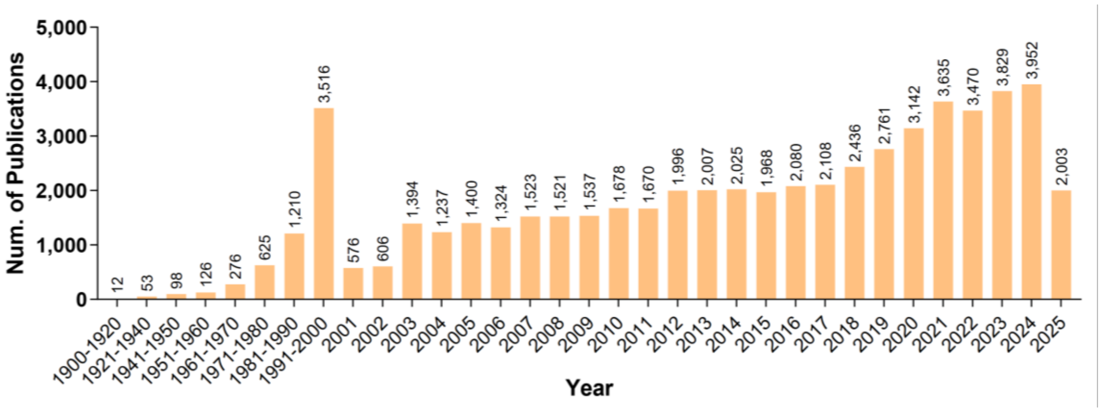

Fig. S2. The number of publications on potato research has increased significantly in recent years.

The dataset was compiled from the Web of Science and PubMed databases using "potato" as a search term in titles or abstracts, with a data retrieval cutoff of June 30, 2025.

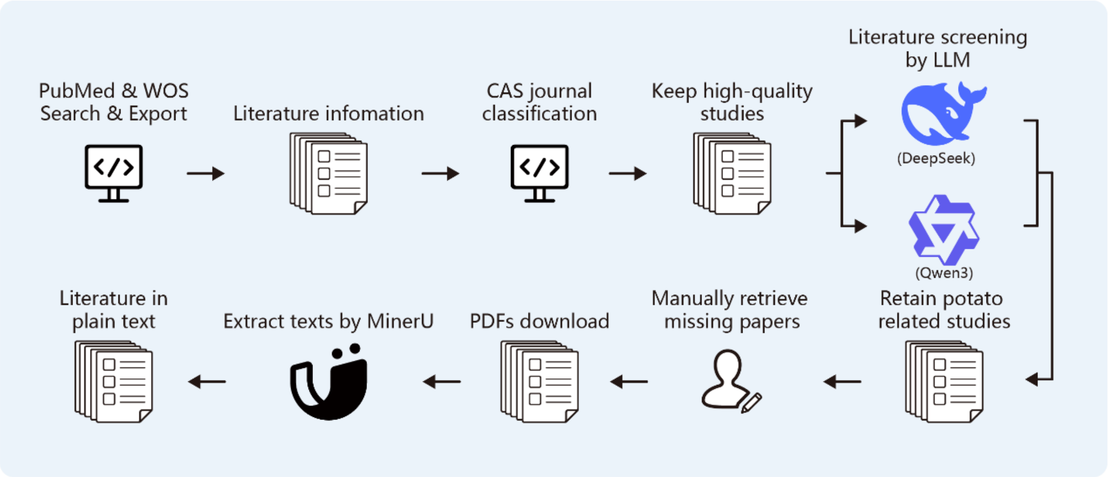

Fig. S3. Pipeline of literature processing.

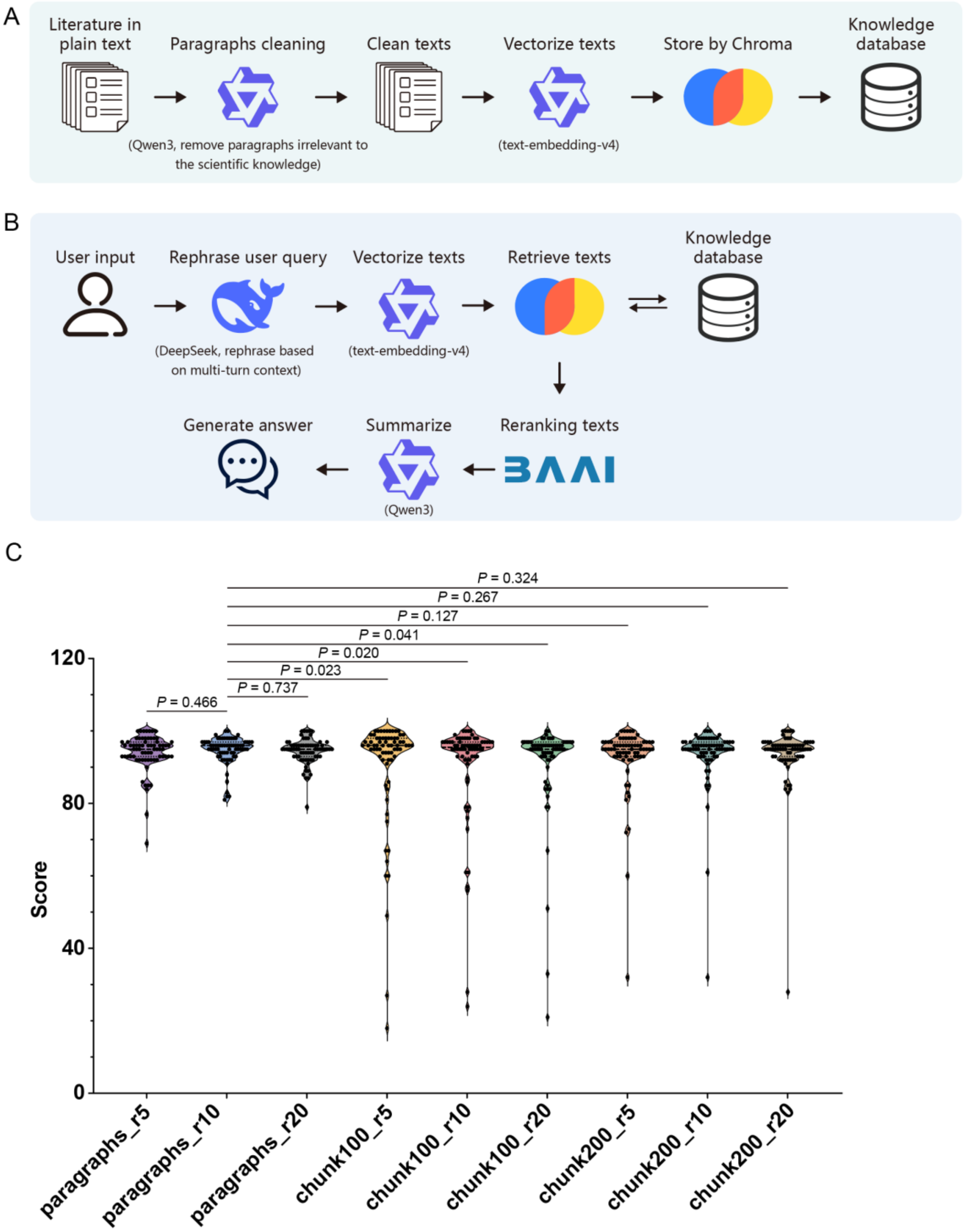

Fig. S4. Development of potato knowledge database.

(A) Pipeline of paper cleaning and vectorization.

(B) Pipeline of query and answer generation for potato knowledge.

(C) Scoring of generation results using different segmentation and fragment retrieval methods. 'Paragraphs', 'chunk100', and 'chunk200' represent segmentation by natural paragraphs, 100-word chunks with 60-word overlap, and 200-word chunks with 120-word overlap, respectively. 'r5', 'r10', and 'r20' represent the retrieval of the top 5, top

10, and top 20 fragments after reranking. Significance was assessed by Student’s t-test.

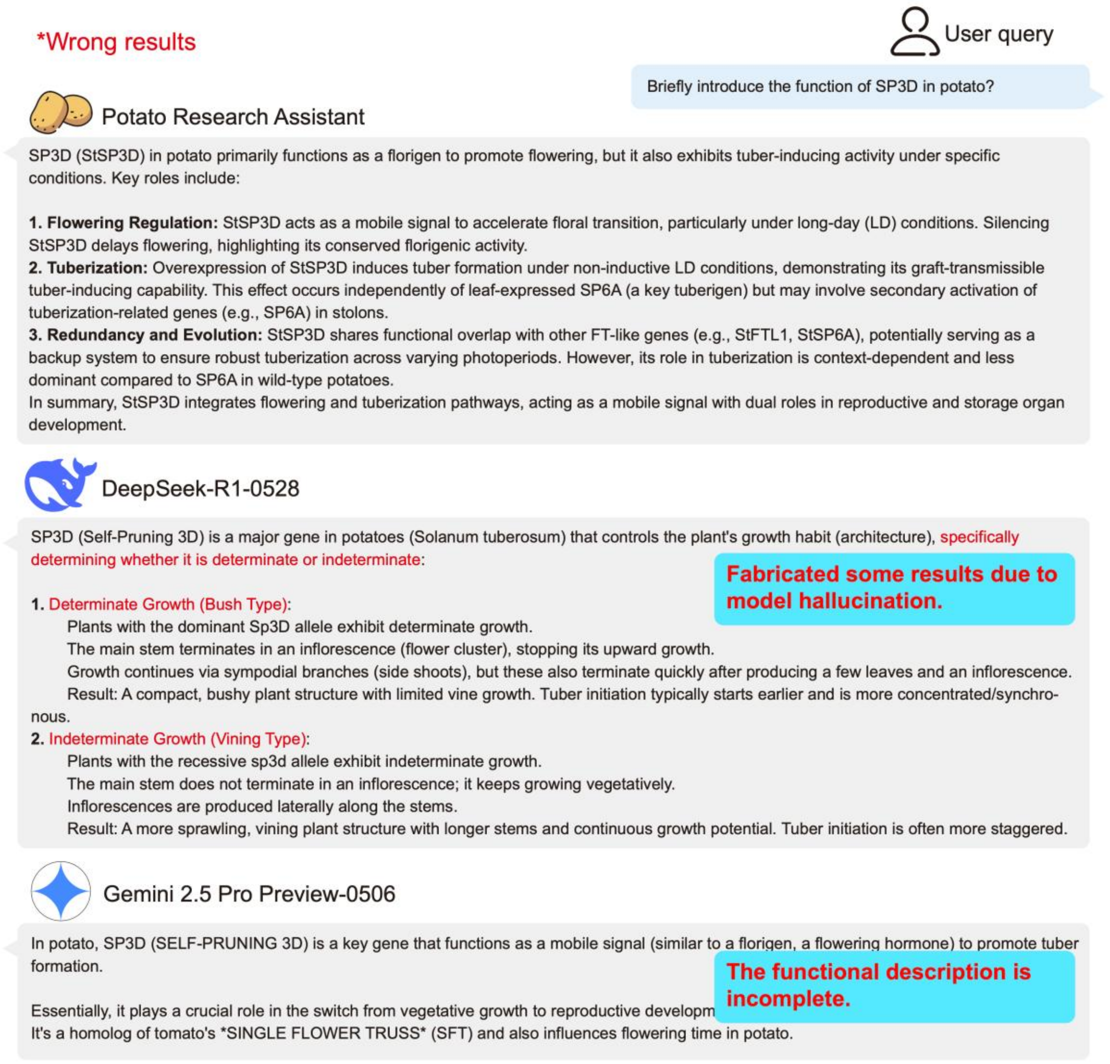

Fig. S5. Comparison of potato knowledge queries between Potato Research Assistant and general LLMs.

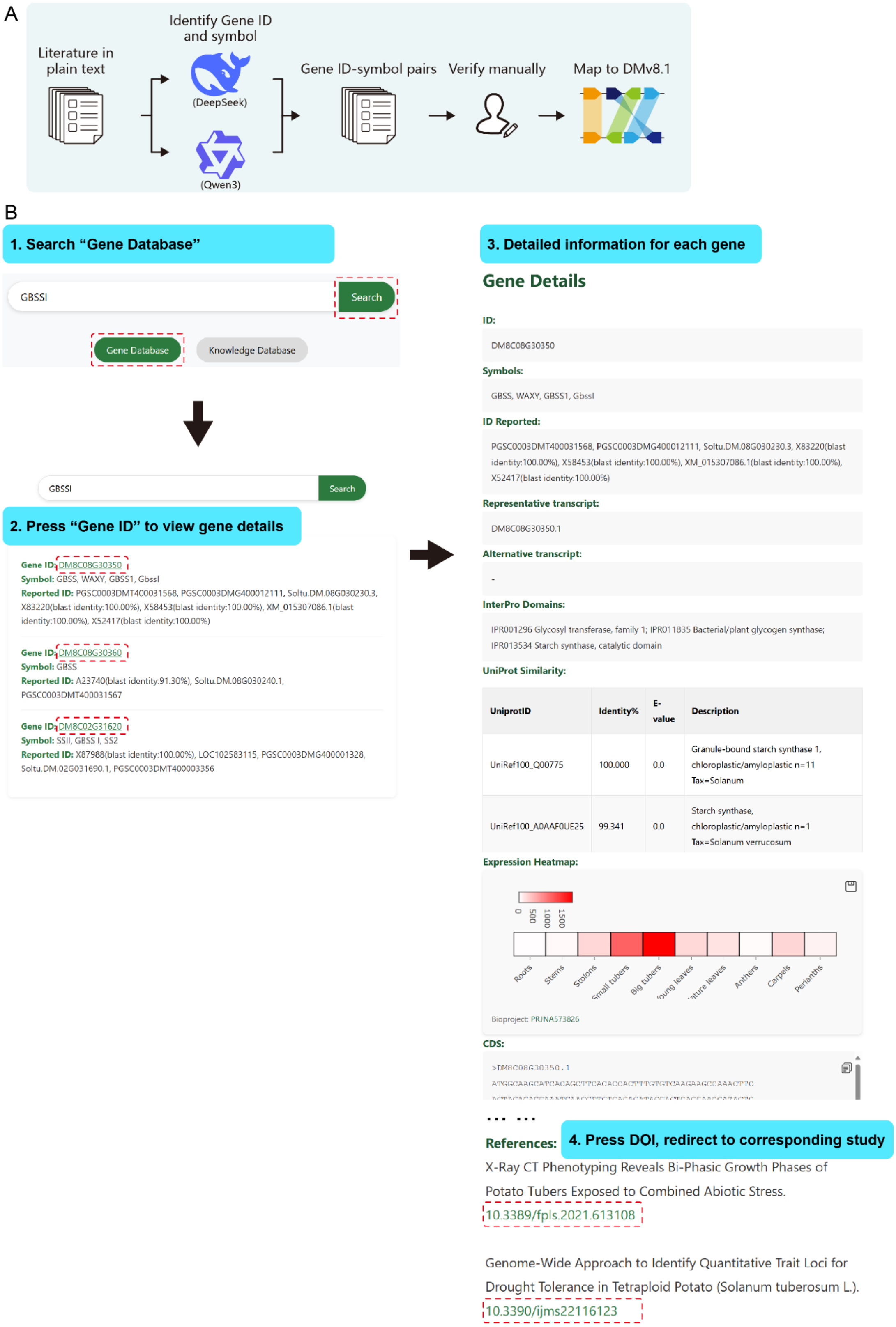

Fig. S6. Construction of potato gene database.

(A) Pipeline of reconciling gene IDs across historical studies.

(B) Function illustration of gene database.

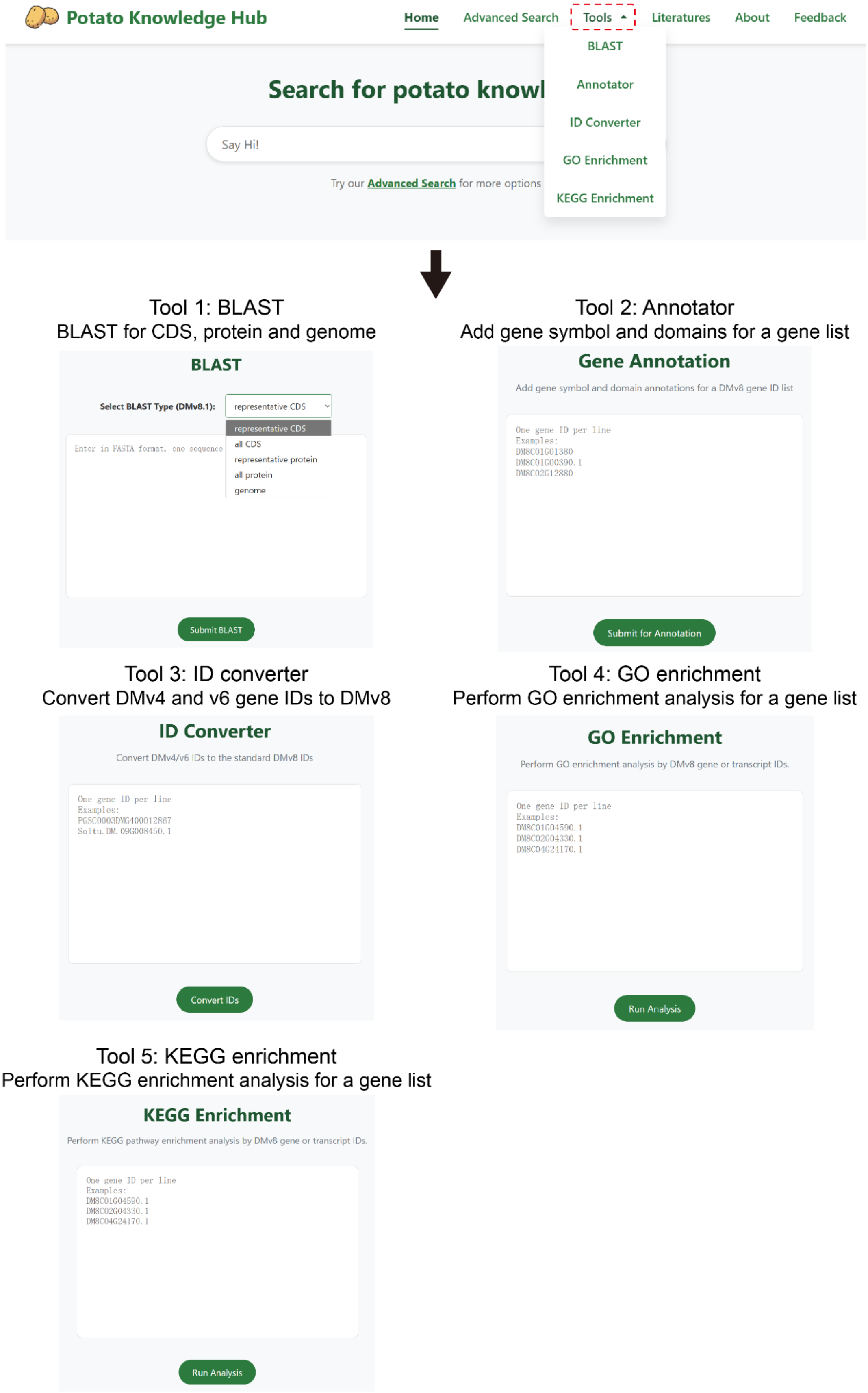

Fig. S7. Toolkit integrated in Potato Knowledge Hub.

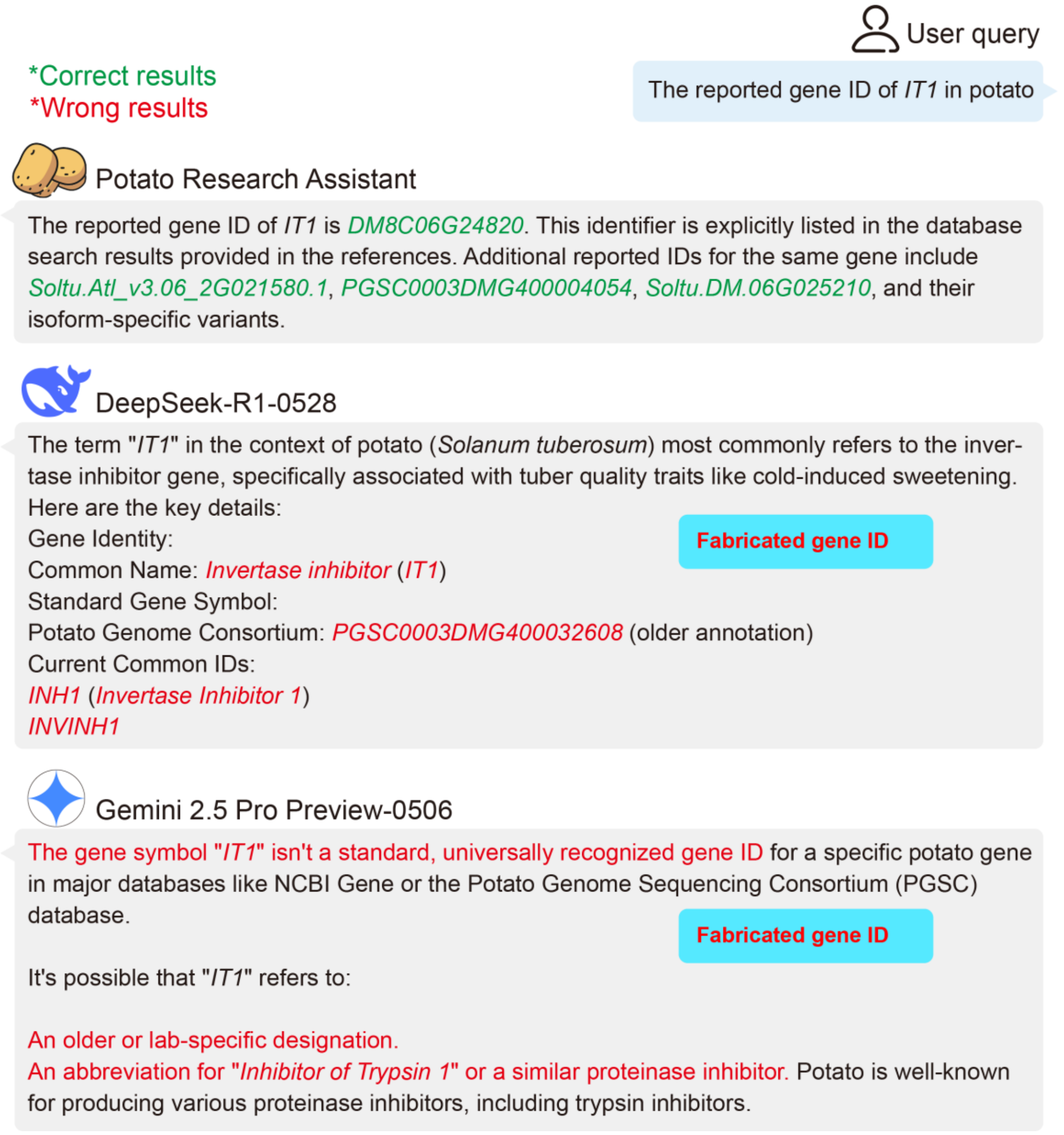

Fig. S8. Comparison of gene detail comprehension between potato research assistant and general LLMs.

The correct results were marked as green fonts, and the fabricated results were marked as red.

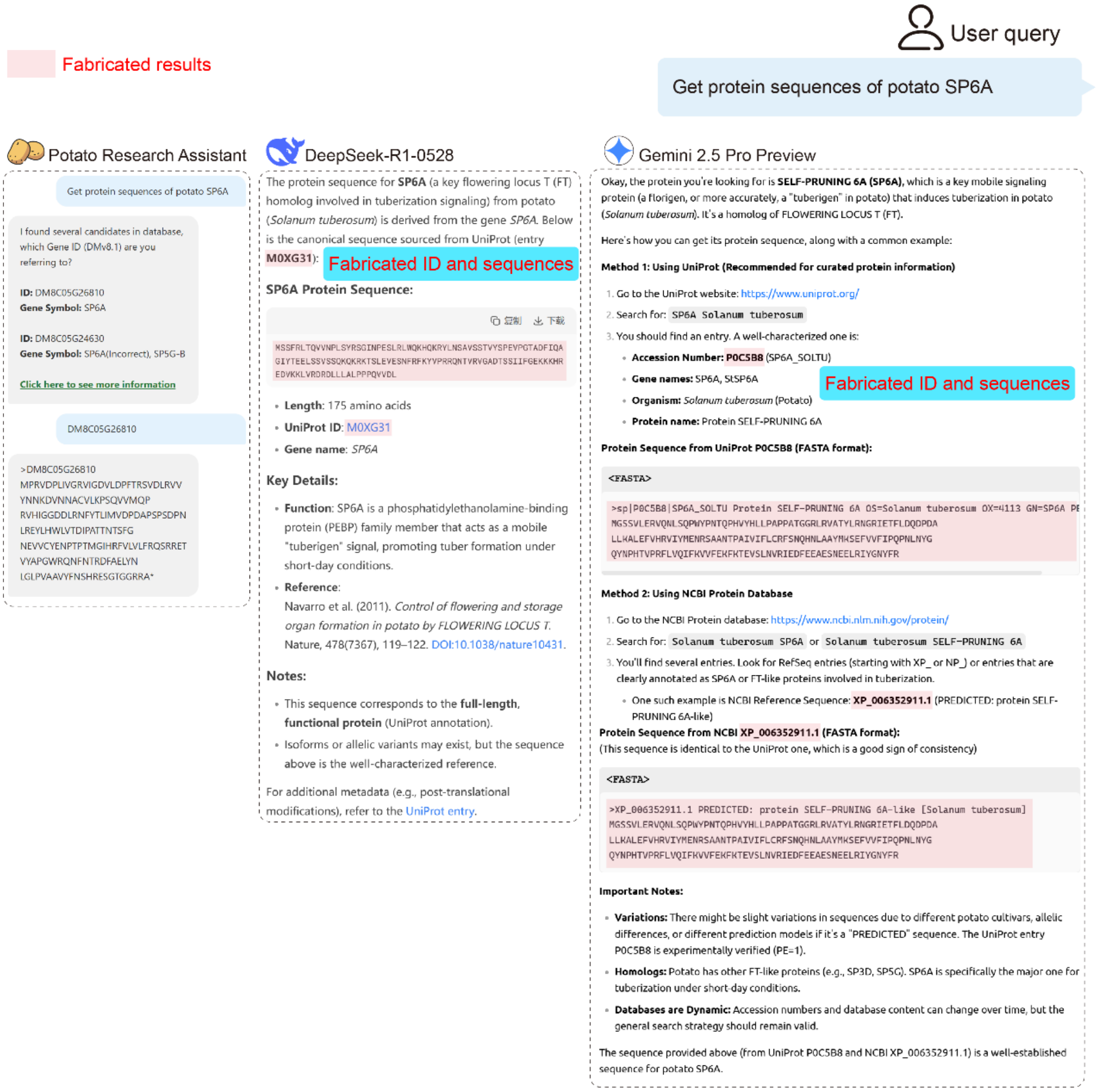

Fig. S9. Comparison of sequence extraction between potato research assistant and general LLMs.

The fabricated gene IDs and sequences generated by general LLMs were marked as pink background.

Supplementary References

Camacho, C., Coulouris, G., Avagyan, V., Ma, N., Papadopoulos, J., Bealer, K., and Madden, T.L. (2009). BLAST+: architecture and applications. BMC Bioinformatics **10**:421. 10.1186/1471-2105-10-421.

Chen, C., Wu, Y., Li, J., Wang, X., Zeng, Z., Xu, J., Liu, Y., Feng, J., Chen, H., He, Y., et al. (2023). TBtools-II: A "one for all, all for one" bioinformatics platform for biological big-data mining. Mol Plant **16**:1733-1742. 10.1016/j.molp.2023.09.010.

Chen, S. (2023). Ultrafast one-pass FASTQ data preprocessing, quality control, and deduplication using fastp. Imeta **2**:e107. 10.1002/imt2.107.

Jones, P., Binns, D., Chang, H.Y., Fraser, M., Li, W., McAnulla, C., McWilliam, H., Maslen, J., Mitchell, A., Nuka, G., et al. (2014). InterProScan 5: genome-scale protein function classification. Bioinformatics **30**:1236-1240. 10.1093/bioinformatics/btu031.

Kim, D., Langmead, B., and Salzberg, S.L. (2015). HISAT: a fast spliced aligner with low memory requirements. Nat Methods **12**:357-360. 10.1038/nmeth.3317.

Krumdick, M., Lovering, C., Reddy, V., Ebner, S., and Tanner, C. (2025). No free labels: Limitations of llm-as-a-judge without human grounding. arXiv preprint arXiv:2503.05061.

Pertea, M., Kim, D., Pertea, G.M., Leek, J.T., and Salzberg, S.L. (2016). Transcript-level expression analysis of RNA-seq experiments with HISAT, StringTie and Ballgown. Nat Protoc **11**:1650-1667. 10.1038/nprot.2016.095.

Suzek, B.E., Wang, Y., Huang, H., McGarvey, P.B., Wu, C.H., and UniProt, C. (2015). UniRef clusters: a comprehensive and scalable alternative for improving sequence similarity searches. Bioinformatics **31**:926-932. 10.1093/bioinformatics/btu739.

Szymanski, A., Ziems, N., Eicher-Miller, H.A., Li, T.J.-J., Jiang, M., and Metoyer, R.A. (2025). Limitations of the llm-as-a-judge approach for evaluating llm outputs in expert knowledge tasks. Proceedings of the 30th International Conference on Intelligent User Interfaces.

Tang, H., Krishnakumar, V., Zeng, X., Xu, Z., Taranto, A., Lomas, J.S., Zhang, Y., Huang, Y., Wang, Y., Yim, W.C., et al. (2024). JCVI: A versatile toolkit for comparative genomics analysis. Imeta **3**:e211. 10.1002/imt2.211.

Wang, B., Xu, C., Zhao, X., Ouyang, L., Wu, F., Zhao, Z., Xu, R., Liu, K., Qu, Y., Shang, F., et al. (2024). MinerU: An open-source solution for precise document content extraction. arXiv preprint

arXiv:2409.18839.

Wickham, H. (2016). ggplot2 : Elegant Graphics for Data Analysis. 2nd ed. Springer-Verlag New York.

Wu, T., Hu, E., Xu, S., Chen, M., Guo, P., Dai, Z., Feng, T., Zhou, L., Tang, W., Zhan, L., et al. (2021). clusterProfiler 4.0: A universal enrichment tool for interpreting omics data. Innovation (Camb) **2**:100141. 10.1016/j.xinn.2021.100141.
